# A Novel Enzyme Portfolio for Red Algal Polysaccharide Degradation in the Marine Bacterium *Paraglaciecola hydrolytica* S66^T^ Encoded in a Sizeable Polysaccharide Utilization Locus

**DOI:** 10.3389/fmicb.2018.00839

**Published:** 2018-05-03

**Authors:** Mikkel Schultz-Johansen, Pernille K. Bech, Rosanna C. Hennessy, Mikkel A. Glaring, Tristan Barbeyron, Mirjam Czjzek, Peter Stougaard

**Affiliations:** ^1^Section for Microbial Ecology and Biotechnology, Department of Plant and Environmental Sciences, University of Copenhagen, Frederiksberg, Denmark; ^2^Sorbonne Université, CNRS, Integrative Biology of Marine Models (LBI2M), Station Biologique de Roscoff (SBR), Roscoff, France

**Keywords:** marine bacteria, carbohydrate-active enzymes, algal polysaccharides, agarase, carrageenase, furcellaran, GH16

## Abstract

Marine microbes are a rich source of enzymes for the degradation of diverse polysaccharides. *Paraglaciecola hydrolytica* S66^T^ is a marine bacterium capable of hydrolyzing polysaccharides found in the cell wall of red macroalgae. In this study, we applied an approach combining genomic mining with functional analysis to uncover the potential of this bacterium to produce enzymes for the hydrolysis of complex marine polysaccharides. A special feature of *P. hydrolytica* S66^T^ is the presence of a large genomic region harboring an array of carbohydrate-active enzymes (CAZymes) notably agarases and carrageenases. Based on a first functional characterization combined with a comparative sequence analysis, we confirmed the enzymatic activities of several enzymes required for red algal polysaccharide degradation by the bacterium. In particular, we report for the first time, the discovery of novel enzyme activities targeting furcellaran, a hybrid carrageenan containing both β-carrageenan and κ/β-carrageenan motifs. Some of these enzymes represent a new subfamily within the CAZy classification. From the combined analyses, we propose models for the complete degradation of agar and κ/β-type carrageenan by *P. hydrolytica* S66^T^. The novel enzymes described here may find value in new bio-based industries and advance our understanding of the mechanisms responsible for recycling of red algal polysaccharides in marine ecosystems.

## Introduction

Macroalgae contain complex and diverse polysaccharides (e.g., agars, carrageenans, alginates, and fucoidans), which constitute a carbon source for a range of marine bacteria (Martin et al., 2014). Hence, understanding the bacterial mechanisms for polysaccharide degradation is crucial to decipher carbon fluxes in marine ecosystems (Azam and Malfatti, 2007; Arnosti, 2011). Bacteria of the phyla *Bacteroidetes* and *Proteobacteria* are often associated with macro- and microalgae and can degrade a range of marine polymeric carbon (Martin et al., 2015). Community-based studies have shown that *Bacteroidetes* are the first taxon to respond to algal blooms (Teeling et al., 2012, 2016), followed later by *Alpha-* and *Gammaproteobacteria*. Accordingly, *Bacteroidetes* generally encode a larger enzymatic potential for carbohydrate degradation than other bacterial phyla (Barbeyron et al., 2016b). These Carbohydrate Active enZymes (CAZymes) often cluster in polysaccharide utilization loci (PULs), which by definition contain the entire capacities for sensing, degradation, transport, and metabolism of a given polysaccharide (Grondin et al., 2017). In *Bacteroidetes*, PULs have traditionally been recognized by the presence of tandem SusC/SusD-like proteins encoding a TonB-dependent transporter and a carbohydrate-binding lipoprotein, respectively (Martens et al., 2011; Terrapon and Henrissat, 2014). More recent results show that the PUL paradigm can be extended and also covers degradation systems lacking SusC/SusD pairs (Ficko-Blean et al., 2017; Grondin et al., 2017); e.g., those present in *Gammaproteobacteria* (Neumann et al., 2015).

Degradation of various algal polysaccharides such as alginate, agar, and carrageenan have been investigated through genomic and functional analysis, in particular, of the marine Flavobacterium *Zobellia galactanivorans* (Hehemann et al., 2012; Thomas et al., 2012, 2017; Barbeyron et al., 2016b; Ficko-Blean et al., 2017). Likewise, similar enzyme systems have been described in other *Bacteroidetes* members such as *Gramella forsetii* (Bauer et al., 2006; Kabisch et al., 2014), *Formosa agariphila* (Mann et al., 2013), and *Polaribacter* spp. (Xing et al., 2015). *Gammaproteobacteria* have also been connected to polymer degradation. Mesocosm studies have indicated that members of the *Alteromonadaceae* family dominate the bacterial community during algal polysaccharide degradation in some ecological niches (Wietz et al., 2015; von Scheibner et al., 2017). For instance, *Alteromonas macleodii* was found to be abundant during alginate degradation (Wietz et al., 2015) and subsequent studies have verified the presence of alginate utilization systems in some ecotypes (Neumann et al., 2015). Likewise, *Glaciecola agarilytica* dominated an agar-degrading community (Wietz et al., 2015) in accordance with the agarolytic phenotype reported for this bacterium (Yong et al., 2007). Several other genera within the *Alteromonadaceae* family have been connected with agarolytic activities such as *Agarivorans* (Kim et al., 2016), *Gayadomonas* (Chi et al., 2013), and *Catenovulum* (Yan et al., 2011), however, little is known about the responsible enzymatic systems within these taxa. *Paraglaciecola hydrolytica* S66^T^ represents a newly isolated *Gammaproteobacterium* of the *Alteromonadaceae* family, which has the ability to degrade both agar and carrageenan (Bech et al., 2017). These abilities have been highlighted by the detection of various genomic systems for polysaccharide degradation (Schultz-Johansen et al., 2016). More insight into the enzymatic machineries of these taxonomic groups may result in a deeper understanding of their role in marine ecosystems.

Agars and carrageenans are major cell wall polysaccharides in different phyla of red macroalgae. In their simplest forms, these polysaccharides are composed of a backbone built from disaccharide blocks (*-bioses*, Supplementary Figure 1) of D-galactose and 3,6-anhydrogalactose (L-AHG in agar and D-AHG in carrageenan) and the resulting galactan backbone is termed agarose and β-carrageenan, respectively (Knutsen et al., 1994). However, the galactan backbone often adapts a diverse range of modifications including sulfation, methylation, and pyruvation (Usov, 2011). For example, in agar from *Porphyra* spp. L-AHG is replaced by L-galactose-6-sulfate; a polymer also known as porphyran (Anderson and Rees, 1965). Likewise, different types of carrageenans have been defined, based on the position and number of sulfate groups along the galactan backbone, the presence of which influences the rheological properties and industrial utilization (Campo et al., 2009); the most common types are κ- and ι-carrageenans. While industrially exploited carrageenophyte red algae contain carrageenans with a majority of sulfated κ- and ι-type (*Kappaphycus* and *Eucheuma* spp.), most natural carrageenans are complex in structure, with varying compositional patterns of disaccharide building blocks along the polymeric chain. For example, furcellaran (extracted from *Furcellaria lumbricalis*) is a common name for hybrid carrageenan composed mainly of κ- and β-type disaccharide motifs (Knutsen et al., 1990; Yang et al., 2011).

According to the CAZy database (Lombard et al., 2014), enzymes hydrolyzing agar belong to glycoside hydrolase (GH) families GH16, GH50, GH86, GH96, GH117, and GH118, whereas carrageenases are found in families GH16, GH82, GH127, GH129, and GH150 (Michel et al., 2006; Martin et al., 2014; Ficko-Blean et al., 2017). In addition, auxiliary enzymes such as sulfatases may assist in the degradation of sulfated algal polysaccharides (Helbert, 2017).

The identification of PULs has been widely used in genome-based studies to predict algal polysaccharide degradation in marine bacteria (Mann et al., 2013; Sun et al., 2016; Zhu et al., 2016; Gao et al., 2017). While such analyses give overall hints about carbohydrate metabolism, the lack of characterized close homologs for the individual proteins makes it difficult to predict the fine substrate specificities. Thus, mining of PULs combined with experimental validation (functional screening) are required to advance our understanding of substrate diversities and the detailed mechanisms used by microbes for the breakdown of complex carbohydrates. Indeed, such an approach has successfully been used for the discovery of new enzyme activities targeting marine polysaccharides (Hehemann et al., 2010; Rebuffet et al., 2011; Foran et al., 2017).

Besides the understanding of their important biological roles, oligosaccharides obtained from algal polysaccharides have recently received increased interest due to their biomedical potential (Hu et al., 2006; Enoki et al., 2012; Morya et al., 2012; Kalitnik et al., 2016). In this context, novel enzymes are important for advancing the bioconversion of algae into biologically active compounds with potential for industrial applications. In addition, the discovery of enzymes having novel activities can serve as tools for structural elucidation of complex carbohydrates (Aarstad et al., 2012).

In this study, we have investigated enzymes involved in red algal degradation by *P. hydrolytica* S66^T^ and we provide tentative models for the catabolism of agar and carrageenan in this marine bacterium. In line with earlier studies we demonstrate that novel enzyme specificities can be discovered from PULs containing unannotated genes or distant homologs to characterized CAZymes. In particular, we report the finding of hitherto unknown furcellaranase-active enzymes, which are likely to represent new GH families or subfamilies within the CAZy classification. The novel enzymes described here may find value in new bio-based industries and advance our understanding of the mechanisms responsible for recycling of red algal polysaccharides in marine ecosystems.

## Materials and Methods

### Data Availability

*P. hydrolytica* S66^T^ was isolated and characterized in a previous study (Bech et al., 2017). The draft genome sequence of *P. hydrolytica* S66^T^ is available from Genbank, acc. no. LSNE00000000 (Schultz-Johansen et al., 2016). The results presented here are based on protein predictions and locus tags generated by RAST (Aziz et al., 2008), and are accessible on the RAST guest account under Genome ID 89404.4^1^ (using login and password ‘guest’). For convenience the RAST generated locus tag prefix (fig|89404.4.peg.) was renamed to ‘Ph’ in the current study.

### Sequence Analyses

Carbohydrate active enzymes and sulfatases were identified in the *P. hydrolytica* S66^T^ genome using Hidden Markov Model searches against local versions of the dbCAN 5.0 (Yin et al., 2012) and Pfam (Finn et al., 2016) databases. Predicted protein sequences were compared with homologs at NCBI using BLASTp searches against the PDB and nr databases. Sulfatase homologs were also compared in the SulfAtlas database^2^ using the built-in BLAST function (Barbeyron et al., 2016a). Manual comparison and synteny of bacterial genomes was assessed using the MaGe interface^3^ on the MicroScope platform (Vallenet et al., 2009). N-terminal secretion signals were predicted using SignalP 4.1 (Petersen et al., 2011) and LipoP 1.0 (Juncker et al., 2003). Multiple alignments and phylogenetic trees were constructed using MEGA6 (Tamura et al., 2013). The integrated ClustalW algorithm was used for aligning GH16 protein sequences. The alignment was manually inspected and then used to construct an unrooted maximum likelihood phylogenetic tree based on 1,000 bootstrap iterations.

### Gene Cloning and Production of Recombinant Enzymes for Functional Screening

Oligonucleotides used in this study are listed in Supplementary Table 1. In general, the primers were designed to target the full gene sequence without any predicted signal peptides. The gene encoding Ph1586 was predicted from an ORF of ∼5 kb and consequently only the proposed catalytic region was PCR amplified. Genomic DNA (gDNA) was extracted from *P. hydrolytica* S66^T^ as described before (Schultz-Johansen et al., 2016) and used as template DNA in PCR reactions. PCRs of enzyme encoding genes were carried out with Phusion DNA polymerase (Novagen) or, for reactions with uracil-containing primers, the *Pfu*-X7 polymerase (Nørholm, 2010), kindly provided by Prof. Halkier, University of Copenhagen. PCR products were purified using the QIAquick PCR purification Kit (Qiagen, Germany). The Ph1631 gene construct was cloned into the *Nde*I and *Xho*I sites of a pET22b vector (Novagen). The remaining gene constructs were inserted into vectors (pET9a-USER-1 and pET9a-USER-2) modified for USER^TM^ cloning according to the procedures described by Nour-Eldin et al. (2010). In brief, to construct the pET9a-USER-1 vector, a gene cassette was assembled from oligonucleotides (Supplementary Table 1) and cloned into the *Nde*I and *Bam*HI sites of a linearized pET9a vector (Novagen). The resulting pET9a-USER-1 featured a USER compatible cloning cassette in addition to a C-terminal octa-histidine tag, and was linearized with *Pac*I and nt.*Bbv*CI prior to cloning. The pET9a-USER-2 vector was generated as a ‘USER fusion’ product (Geu-Flores et al., 2007) by PCR amplification of pET9a-USER-1 using the primers specified in Supplementary Table 1. Assembly of purified PCRs and pET9a-USER vectors was carried out with USER^TM^ enzyme (NEB) as previously described (Nour-Eldin et al., 2010).

Vector constructs were transformed into *E. coli* TOP10 cells and incubated overnight at 37°C on LB agar with ampicillin (100 μg/mL) for pET22b and kanamycin (50 μg/mL) for pET-USER constructs. Recombinant colonies were picked from plates and cultivated in LB broth with appropriate antibiotics at 37°C overnight with shaking. Recombinant plasmids were purified using the MiniPrep Plasmid DNA Purification Kit (Qiagen) and verified by DNA sequencing before production of recombinant enzymes.

For enzyme production, recombinant plasmids were transformed into *E. coli* BL21(DE3) cells (as described above). Single colonies were picked from plates and cultivated in 20 mL ZYP-5052 autoinduction medium (Studier, 2005) supplemented with antibiotics for 3 days at 20°C with shaking. Cells were collected by centrifugation and resuspended in 0.5 mL lysis buffer (50 mM HEPES, pH = 8, 100 mM NaCl or 50 mM Tris-HCl, pH = 8, 100 mM NaCl). For Ph1656, Ph1659, Ph1663, and Ph1675, the lysis buffer also contained 5 mM CaCl_2_ and for Ph1664 the lysis buffer contained 300 mM NaCl and 5 mM MgCl_2_. Cell lysis was performed in a FastPrep-24^TM^ 5G bead beater (MP Biomedicals) using glass beads size 212–300 μm/425–600 μm in the ratio 1:1 (Sigma-Aldrich). After cell disruption, the soluble and insoluble protein fractions were separated by centrifugation.

### Enzyme Reactions and Activity Visualization

Crude recombinant cell lysates were screened for activity against algal substrates; agar, κ-carrageenan, ι-carrageenan (Sigma-Aldrich), agarose (Invitrogen, United Kingdom, <0.351% sulfate), porphyran (prepared as described in Hehemann et al., 2010), and furcellaran (Est-Agar, Kärla, Estonia). Agar-type oligosaccharides were obtained from Aglyco (Beijing, China) and L-AHG used as a standard was a kind gift from Prof. Kyoung Heon Kim (Korea University, South Korea). Carrageenan-type oligosaccharides were obtained from Dextra (Reading, United Kingdom).

For a 100 μL enzyme reaction, 10 μL crude cell lysate was added to polysaccharides (final concentration 0.05% w/v) or oligosaccharides (2 μg/μL) in the corresponding cell lysis buffer. Samples were incubated at 25°C overnight and degradation products from enzymatic reactions were analyzed by thin layer chromatography (TLC) or fluorophore-assisted-carbohydrate-electrophoresis (FACE). TLC was performed on silica gels 60 F254 (Merck) using *n*-butanol/acetic acid/water (2:1:1 v/v/v) as a running solvent and plates were developed on a hot plate after spraying with orcinol. FACE was performed using labeling fluorophores 8-aminonaphthalene-1,3,6-trisulfonate (ANTS) or 2-aminoacridone (AMAC) as described before (Starr et al., 1996; Hehemann et al., 2010). Briefly, enzyme reactions were dried in a speed-vac centrifuge and the pellets were dissolved in 2 μL ANTS solution (0.15 M ANTS in acetic acid/water (3:17, v/v) and 5 μL freshly prepared 1 M sodium cyanoborohydride in DMSO) or AMAC solution (0.1 M AMAC in acetic acid/DMSO (3:17, v/v) and 5 μL freshly prepared 1 M sodium cyanoborohydride in water). After derivatization at 37°C for 20 h, the samples were subjected to electrophoresis through a 6% stacking and 27% running polyacrylamide gel at 4°C for 30 min at 15 mA followed by 30 mA for 2 h. Fluorescently labeled oligosaccharides in the polyacrylamide gel were visualized under UV light.

### RNA Extraction and Reverse Transcription PCR (RT-PCR)

For RNA extraction, strain S66^T^ was grown in Minimal Marine Medium (Thomas et al., 2011), supplemented with 2 g/L agarose, agar, porphyran, or glucose that had been sterilized by autoclaving. Cells were cultivated at 20°C for 5 days with shaking (200 rpm) and total RNA was extracted using the ZR Fungal/Bacterial RNA MiniPrep^TM^ kit according to the manufacturer’s instructions (Zymo Research, United States). RNA purity and concentration were determined using a Qubit^®^ RNA HR Assay Kit (Invitrogen) and RNA quality was assessed by agarose gel electrophoresis. Contaminating DNA was removed using the DNA-free^TM^ DNA removal kit (Ambion) according to the manufacturer’s instructions. RNA was checked for gDNA contamination by PCR prior to performing cDNA synthesis. Reverse transcription was performed with 50 ng total RNA of each sample using a GoScript^®^ Reverse Transcription System (Promega) using non-sequence specific primers according to manufacturer instructions. The obtained cDNA was stored at -20°C and used as template in subsequent PCR reactions. Target-specific primers were designed using CLC (Main Workbench) software (CLC BIO, Aarhus, Denmark) to amplify selected genes encoding enzymes predicted to be involved in polysaccharide degradation (Supplementary Table 2). PCRs were performed using the GoTag^®^ DNA polymerase (Promega) with 10 μM of each primer and 1 μL RNA or gDNA (control) in a total reaction volume of 24 μL using the following cycling parameters: 98°C for 2 min, 30 cycles of 10 s at 98°C, 30 s at 55°C, and 30 s at 72°C, followed by a final step at 72°C for 5 min. PCR products (4 μL) were visualized on agarose gels and semi-quantitatively assessed. All experiments were performed with biological triplicates.

## Results and Discussion

### The Potential for Polysaccharide Degradation by *P. hydrolytica* S66^T^

*P. hydrolytica* S66^T^ (from now on referred to as S66^T^) is capable of degrading several plant- and algal-derived polysaccharides, most notably red algal polysaccharides ranging from low-sulfate containing agarose to sulfated agar, porphyran, and κ-carrageenan (Bech et al., 2017). These phenotypic traits correlate with a preliminary survey of the S66^T^ genome encoding a large number of putative enzymes with predicted functions related to carbohydrate utilization (Schultz-Johansen et al., 2016). In this study, a more extensive analysis of the carbohydrate utilization potential was carried out (Supplementary Data Sheet 1). This revealed a total of 270 CAZy modules in the S66^T^ genome, 188 of which were assigned to protein families involved in carbohydrate degradation, i.e., GHs, polysaccharide lyases (PLs) and carbohydrate esterases (CEs). Notably, the CAZyme potential of S66^T^ exceeded that of another Gammaproteobacterium *P. atlantica*, which is considered a “super-degrader” (Barbeyron et al., 2016b). Additional enzymes included 35 glycosyl transferases (GTs), 11 auxiliary activities (AAs), and 36 unique proteins associated with 46 carbohydrate-binding modules (CBMs). Twelve putative sulfatases were also identified, and except for one candidate (S4 β-lactamase family) these represented various subfamilies within the S1 formylglycine-dependent sulfatases according to the SulfAtlas database (Barbeyron et al., 2016a). The existence of secretion signals in all S1 sulfatases suggests a potential function against external substrates and supports a proposed role in scavenging of sulfated carbohydrates for most of these sulfatases.

A closer investigation of the CAZyme repertoire revealed several candidates belonging to GH families with characterized red algae-specific members: Eight GH16 enzymes (β-agarases, κ-carrageenases, β-porphyranases or glucanases), four GH50 enzymes (β-agarases), one GH82 (ι-carrageenase), two GH86 (β-agarases or β-porphyranases), one GH117 (α-1,3-(3,6-anhydro)-L-galactosidase), and one GH127 (α-1,3-(3,6-anhydro)-D-galactosidase). No representatives from agarolytic families GH96 (α-agarases) or GH118 (β-agarases) were identified.

### All Genes for Red Algal Polysaccharide Degradation Are Organized in a Single, Large Gene Cluster

**FIGURE 1:**
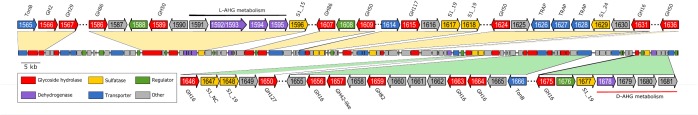
Genomic organization of a ∼167 kb PUL-like region in *P. hydrolytica* S66^T^ encoding the proposed enzyme repertoire for complete degradation of agar and carrageenan. Putative gene functions are color-coded and regions flanking putative GHs are highlighted. Locus tags are indicated on the genes and protein family/type is indicated next to the genes, if available. Black and red bars indicate genes for metabolism of L- and D-AHG, respectively. Agarolytic and carrageenolytic PUL segments are shaded in yellow and green, respectively.

All CAZymes with a potential activity against red algal polysaccharides were clustered in a contiguous genomic region spanning ∼167,000 bp (**Figure 1**). The GH genes in this region co-localized with transporters (TonB and TRAP), sulfatases and dehydrogenases. Despite the absence of SusC/SusD homologs this genomic organization resembled that of PULs in *Bacteroidetes* (Martens et al., 2009). TonB dependent receptors in this region are likely to contribute to oligosaccharide detection and transport in analogy to the bacteroidetal SusC/SusD system (Blanvillain et al., 2007; Thomas et al., 2012; Neumann et al., 2015). Based on sequence-based and functional analyses, at least two overall sub-PULs could be assigned: one dedicated to agar degradation (Ph1566–Ph1636) and the other responsible for carrageenan degradation (Ph1646–Ph1681).

#### Agarolytic Degradation System

Homology searches against the GenBank nr database, using proteins encoded in the PUL-like regions, predominantly identified proteins with uncharacterized functions as the closest relatives. Sequence comparison against characterized proteins from PDB revealed that GHs located in the first part of the PUL-like region (Ph1586–Ph1636) displayed sequence similarity to characterized β-agarases (Ph1631_GH16, Ph1589_GH50, Ph1609_GH50, Ph1624_GH50, and Ph1636_GH50), β-porphyranases (Ph1586_GH86, Ph1607_GH86) and one α-1,3-(3,6-anhydro)-L-galactosidase (Ph1615_GH117) (**Figure 1** and Supplementary Table 3). In the near upstream region, we also identified a candidate homolog to a characterized agarolytic β-galactosidase (Ph1566_GH2). Ph1566_GH2 displayed 40% sequence identity to *Vej*ABG, which has previously been shown to release galactose from the non-reducing end of agaro-oligosaccharides (Lee C.H. et al., 2014). RT-PCR experiments supported an agarolytic role, since the GH genes tested in the range Ph1586–Ph1636 (except Ph1615) were induced in response to agarose and porphyran substrates (Supplementary Figure 2A).

**FIGURE 2:**
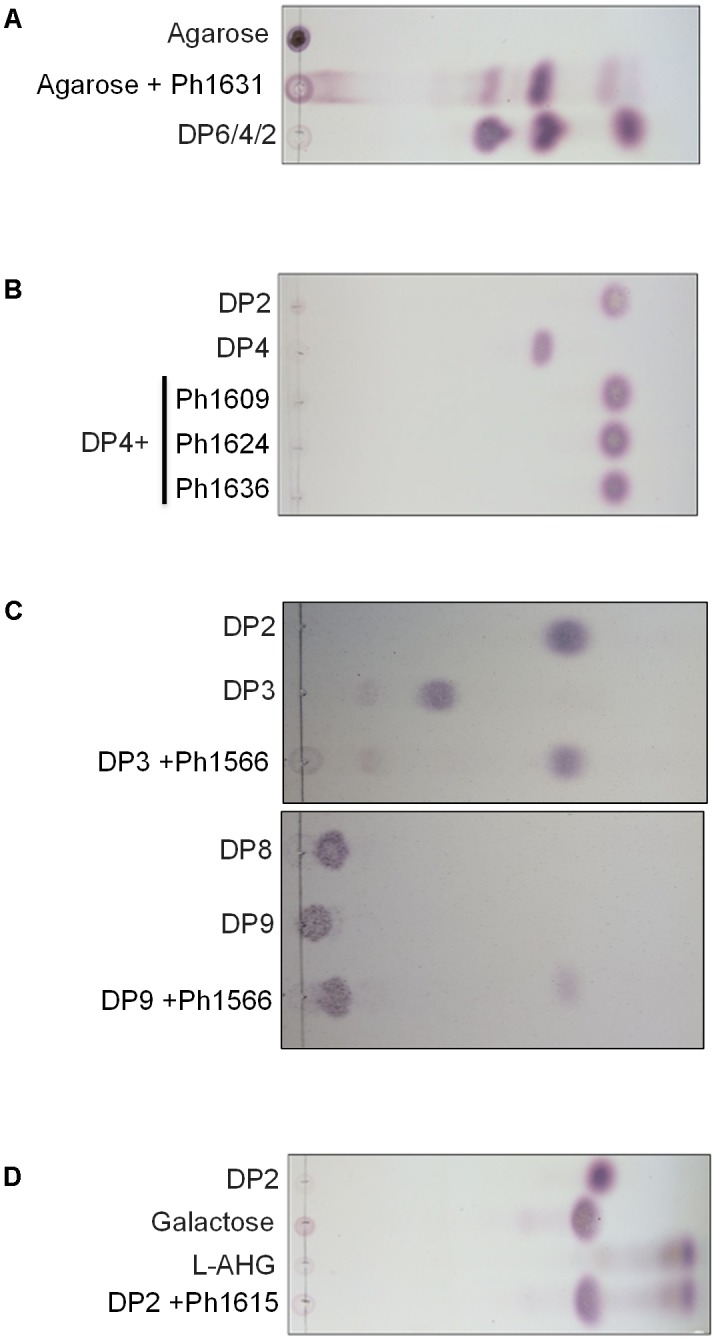
Thin-layer chromatography showing hydrolysis products released from agarose and commercial agar-derived oligosaccharide standards by the action of recombinant enzymes of **(A)** Ph1631 (GH16); **(B)** Ph1609, Ph1624, and Ph1636 (GH50); **(C)** Ph1566 (GH2); **(D)** Ph1615 (GH117). Standards with an even-numbered degree of polymerization (DP) correspond to neoagaro-oligosaccharides, whereas those with an odd-numbered DP correspond to agaro-oligosaccharides.

The agarolytic role of six GHs was confirmed in a preliminary functional characterization (**Table 1**). *E. coli* cell lysates expressing the genes encoding agarolytic GHs were incubated with agar-type substrates and enzymatic degradation was visualized by TLC (**Figure 2**). TLC analysis showed that Ph1631 degraded agarose into primarily neoagarotetraose, with some neoagarobiose and neoagarohexaose also produced (**Figure 2A**). In addition, Ph1631 also displayed activity against porphyran (but only little, compared to agarose) and neoagaro-oligosaccharides, releasing neoagarotetraose and a small amount of neoagarobiose as the major products (data not shown). This suggested that Ph1631 is a GH16 β-agarase most likely exhibiting endolytic activity. Ph1609, Ph1624, and Ph1636 (GH50) showed activity on agar and agarose, as well as on neoagaro-oligosaccharides down to neoagarotetraose. The main hydrolysis products of all three enzymes were neoagarobiose indicating that Ph1609, Ph1624, and Ph1636 represent GH50 exo-β-agarases (**Figure 2B**). Ph1566 (GH2) released galactose from the non-reducing end of odd-numbered agaro-oligosaccharides (**Figure 2C**) but was unable to hydrolyze even-numbered neo-oligosaccharides and lactose (data not shown). This confirmed that Ph1566_GH2 shares the function of *Vej*ABG from *Vibrio* sp. EJY3 which until this date was the only report of an agarolytic GH2 β-galactosidase (Lee C.H. et al., 2014). Finally, Ph1615 (GH117) was able to hydrolyze neoagarobiose yielding L-AHG and galactose confirming that Ph1615 was an α-1,3-(3,6-anhydro)-L-galactosidase (Rebuffet et al., 2011) (**Figure 2D**).

**Table 1:** Functionally verified enzymes encoded within the agarolytic and carrageenolytic PUL-like regions in *P. hydrolytica* S66^T^.

Locus tag	CAZy family	Substrate	Accession no. of closest characterized protein	Percent identity
**Agarolytic activities**		
Ph1566	GH2	Agaro-oligosaccharides ≥ DP3	AEX22320.1; Agarolytic β-galactosidase^1^	40%
Ph1609	GH50	Neoagaro-oligosaccharides ≥ DP4	4BQ2_A; Exo-β-agarase^2^	61%
Ph1615	GH117	Neoagaro-oligosaccharides ≥ DP2	3R4Y_A; α-1,3-(3,6-anhydro)-L-galactosidase^2^	73%
Ph1624	GH50	Neoagaro-oligosaccharides ≥ DP4	4BQ2_A; Exo-β-agarase^2^	47%
Ph1631	GH16	Agarose	3WZ1_A; β-agarase^3^	68%
Ph1636	GH50	Neoagaro-oligosaccharides ≥ DP4	4BQ2_A; Exo-β-agarase^2^	31%
**Carrageenolytic activities**		
Ph1656	GH16 (New subfamily)	Furcellaran	3JUU_A; β-porphyranase^4^	26%
Ph1657	GH42-like	Neocarratetraose monosulfate (β-κ)	5DFA _A; β-galactosidase^5^	18%
Ph1663	GH16 (New subfamily)	Furcellaran	4ATE_A; β-porphyranase^4^	29%
Ph1664	GH16	κ-carrageenan and furcellaran	1DYP_A; κ-carrageenase^6^	40%
Ph1675	GH16 (New subfamily)	Furcellaran-oligosaccharides	4ATE_A; β-porphyranase^4^	27%

*Protein homologs originate from ^1^Vibrio sp. EJY3, ^2^Saccharophagus degradans 2-40^T^, ^3^Microbulbifer thermotolerans JAMB A94^T^, ^4^Zobellia galactanivorans Dsij^T^, ^5^Geobacillus stearothermophilus T6 and ^6^Pseudoalteromonas carrageenovora 9^T^.*

Complete hydrolysis of agar into monomers results in L-AHG and D-galactose. Interestingly, close homologs to characterized enzymes responsible for metabolism of L-AHG (Lee S.B. et al., 2014; Yun et al., 2015) were also identified in the PUL-like cluster (**Figure 1** and Supplementary Table 3). In both *Vibrio* sp. EJY3 and *P. atlantica* T6c, the first two steps in L-AHG metabolism involves an L-AHG dehydrogenase that oxidize L-AHG to AHGA and an L-AHG cycloisomerase that isomerize AHGA into L-KDGal (Lee S.B. et al., 2014; Yun et al., 2015). Homologs of these enzymes (71–87% identity) are also found in S66^T^. In *Vibrio* sp. EJY3, L-KDGal is believed to enter the central metabolism after these two steps (Yun et al., 2015), whereas in *P. atlantica*, L-KDGal is further processed to KDPG by a three-step reaction before entering the Entner–Doudoroff pathway (Lee S.B. et al., 2014). These three latter enzymes have homologs in S66^T^ (49–89% identity) and in addition (except for the KDG kinase), the L-AHG pathways from *P. atlantica* and S66^T^ share complete synteny (Supplementary Figure 3A). Therefore, S66^T^ is most likely to metabolize L-AHG according to the enzymatic pathway reported for *P. atlantica* T6c (Lee S.B. et al., 2014).

**FIGURE 3:**
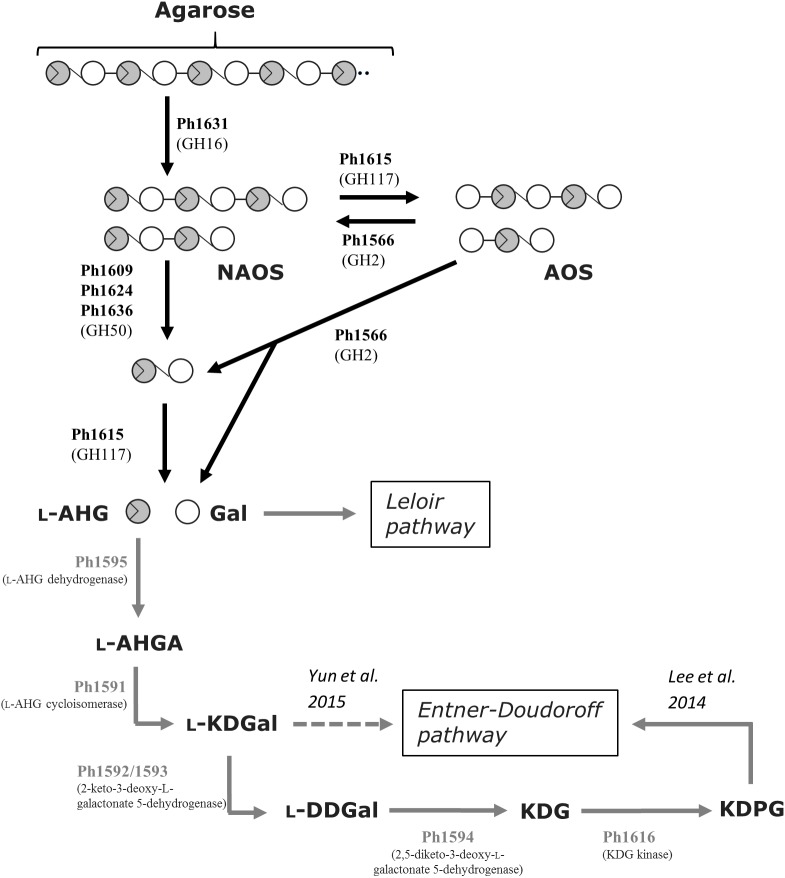
Tentative pathway for agarose degradation in *P. hydrolytica* S66^T^. Enzymes are indicated at each reaction step together with CAZy family if applicable. Solid arrows represent the proposed pathway of *P. hydrolytica* S66^T^ based on functional (black) and bioinformatics (gray) analyses. The dashed arrow represents an alternative catabolic fate of L-KDGal as reported elsewhere. NAOS, neoagaro-oligosaccharides; AOS, agaro-oligosaccharides; L-AHG, 3,6-anhydro-L-galactose; Gal, D-galactose; L-AHGA, 3,6-anhydro-L-galactonate; L-KDGal, 2-keto-3-deoxy-L-galactonate; L-DDGal, 2,5-diketo-3-deoxy-L-galactonate; KDG, 2-keto-3-deoxy-D-gluconate; KDPG, 2-keto-3-deoxy-6-phospho-D-gluconate.

Based on the functional validated enzymes and sequence-based analyses, we can account for the complete metabolic agarose pathway in S66^T^ (**Figure 3**). At least, the agarolytic system includes an endo-acting GH16 β-agarase (Ph1631) that produces neoagaro-oligosaccharides, which are further processed by GH50 β-agarases (Ph1609, Ph1624, and Ph1636) into neoagarobiose or alternatively by a GH117 α-1,3-(3,6-anhydro)-L-galactosidase (Ph1615) into agaro-oligosaccharides. Eventually, the synergistic action of a GH2 agarolytic β-galactosidase (Ph1566) and the α-1,3-(3,6-anhydro)-L-galactosidase (Ph1615) will result in the formation of L-AHG and D-galactose monosaccharides. Whereas, galactose presumably enters directly into the Leloir pathway, L-AHG in S66^T^ is further processed by a L-AHG dehydrogenase (Ph1595) into L-AHGA, L-AHG cycloisomerase (Ph1591) into L-KDGal, 2-keto-3-deoxy-L-galactonate-5-dehydrogenase (Ph1592/93) into L-DDGal, 2,5-diketo-L-galactonate-5-dehydrogenase (Ph1694) into KDG, which is phosphorylated by KDG kinase (Ph1616) and enters the Entner–Doudoroff pathway.

#### Carrageenolytic Degradation System and Discovery of a New GH16 Subfamily

**FIGURE 4:**
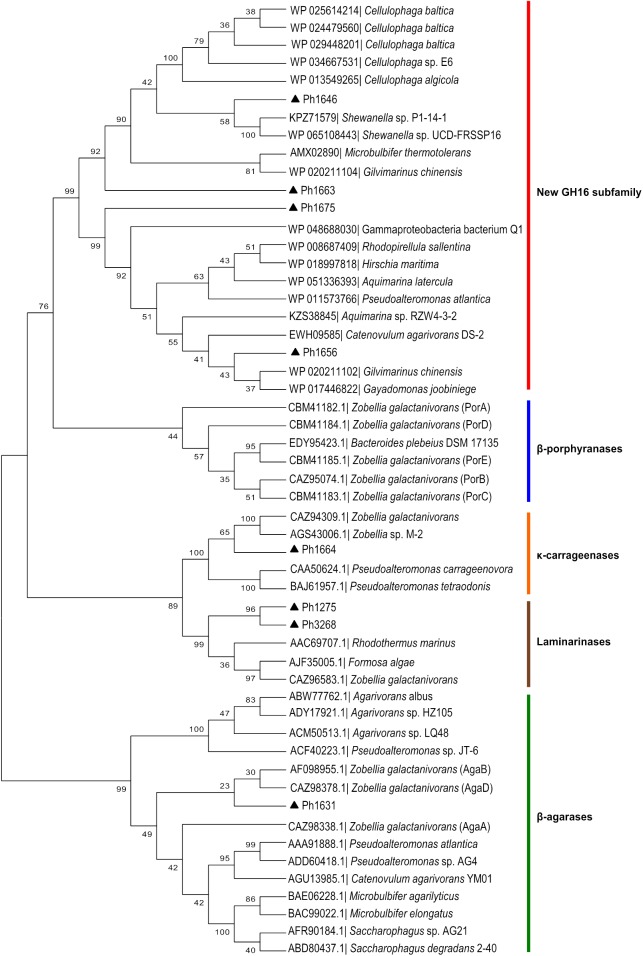
Sequence-based relationship of GH16 family proteins from *P. hydrolytica* S66^T^ (black triangles) and homologs obtained from public databases. Ph1646, Ph1656, Ph1663, and Ph1675 are part of a separate phylogenetic clade forming a new GH16 subfamily. Accession number and taxonomy is indicated for each protein. The β-agarases, β-porphyrases, κ-carrageenases, and laminarinases represent characterized enzymes according to the CAZy database.

**FIGURE 5:**
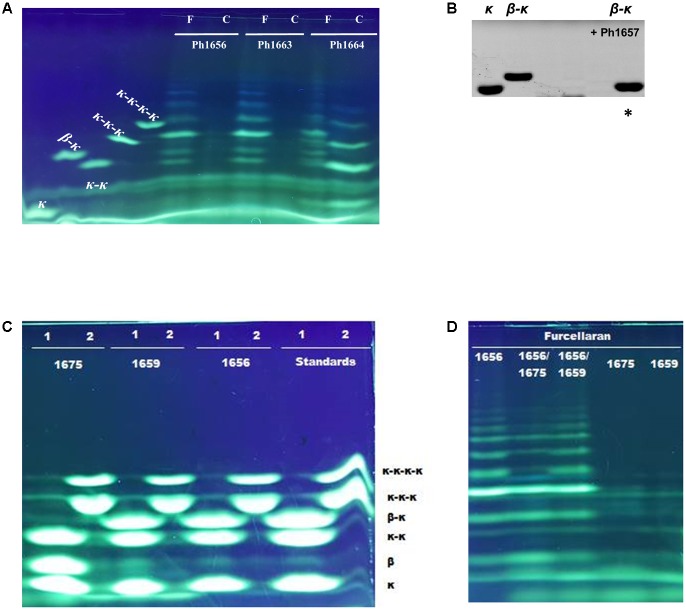
FACE of hydrolysis products released by carrageenolytic enzymes from *P. hydrolytica* S66^T^. Samples were prepared using ANTS **(A,C,D)** or AMAC **(B)** fluorophore. **(A)** Ph1664 degrades both kappa-carrageenan (C) and furcellaran (F), whereas Ph1656 and Ph1663 are specific for furcellaran. **(B)** Ph1657 degrades β-κ tetrasaccharide into the resulting disaccharides β and κ. ^∗^(Migration of non-sulfated β is not observed since uncharged AMAC was used as labeling fluorophore). **(C)** Ph1675 degrades β-κ tetrasaccharide into the resulting disaccharides β and κ. Oligosaccharides standards were applied as substrates in a mix containing κ, κ-κ and β-κ (1) or κ-κ-κ and κ-κ-κ-κ (2). **(D)** Ph1675 is inactive on furcellaran, but degrades the furcellaran-oligosaccharides released by Ph1656.

The function of GHs in the region Ph1646–Ph1681 in the PUL-like cluster was not completely obvious at first. Although the identification of a putative κ-carrageenase (Ph1664_GH16), ι-carrageenase (Ph1659_GH82), and α-1,3-(3,6-anhydro)-D-galactosidase (Ph1650_GH127) immediately suggested a role in carrageenan degradation, we identified in the same region four candidate β-porphyranases (Ph1646_GH16, Ph1656_GH16, Ph1663_GH16, and Ph1675_GH16) with 26–33% sequence identity to characterized homologs (**Figure 1** and Supplementary Table 3). Ambiguous induction patterns were observed for these genes in S66^T^ cells grown on agar-type substrates, and in general for genes in the range Ph1646–Ph1675 (Supplementary Figure 2B). No data was obtained from cells grown on carrageenan due to gel formation on filter membranes used for RNA extraction. The apparent co-localization of porphyranases and carrageenases was puzzling since their corresponding substrates are occurring in different red algae (agarophytes and carrageenophytes), respectively. Therefore, these four enzymes were subjected to further analyses. Members of the GH16 family represent a wide range of enzyme activities and consequently a phylogenetic tree was established in order to compare their sequence-based relationship with characterized homologs (**Figure 4**). Indeed, Ph1646, Ph1656, Ph1663, and Ph1675 were unrelated to known GH16 β-porphyranases, but more intriguingly these four enzymes formed a separate clade, distant to all other characterized GH16 members. These enzymes are most likely to represent a new GH16 subfamily, with not yet defined substrate specificity. In addition, this functionally uncharacterized group also contains other homologs identified by BLASTp searches, all of which derive from marine bacteria. Simultaneously, the GH16 phylogenetic analysis supported the functional validation of Ph1631 as a β-agarase and Ph1664 as a proposed κ-carrageenase. The two remaining GH16s identified in the S66^T^ genome (Ph1275 and Ph3268) affiliated with characterized laminarinases (hydrolyzing brown algal storage glucan). These were found in genomic localizations remote to the agarolytic and carrageenolytic clusters. The confident phylogenetic assignment of Ph1664 as a κ-carrageenase and the co-existence of a GH127 α-1,3-(3,6-anhydro)-D-galactosidase homolog (sharing 60–62% identity to the characterized counterparts ZGAL_3148 and ZGAL_3150 from *Z. galactanivorans*), was a first hint toward a potential substrate for the newly discovered group of GH16s. Whereas, GH16 κ-carrageenases initiate the depolymerization of κ-carrageenan (Michel et al., 2006), the recently discovered GH127 α-1,3-(3,6-anhydro)-D-galactosidases from *Z. galactanivorans* are capable of releasing D-AHG from the non-reducing end of β-neocarrabiose saccharides, as the last step in carrageenan depolymerization (Ficko-Blean et al., 2017). Likewise, the substrate for Ph1646, Ph1656, Ph1663, and Ph1675 could be carrageenans containing κ- or β-carrabiose motifs. To test this hypothesis, an additional activity screening was carried out using the recombinant cell lysates obtained from *E. coli* clones expressing the new GH16 enzymes. Crude cell lysates were incubated with κ-carrageenan and furcellaran (a carrageenan that consists of both κ- and β-carrabiose motifs), and substrate degradation was visualized by FACE (**Figures 5A–D**).

Interestingly, cell lysates of recombinant Ph1656 and Ph1663 showed endolytic activity on the κ/β hybrid carrageenan, but not with κ-carrageenan (**Figure 5A**). This study is the first to describe such a GH16 ‘β-carrageenase’ activity. For comparison, we also cloned and tested a recombinant version of Ph1664, which was a predicted κ-carrageenase. In contrast to the ‘β-carrageenases,’ Ph1664 degraded both furcellaran and κ-carrageenan releasing hydrolysis products corresponding to neo-κ-carrabiose (κ), neo-κ-carratetraose (κ-κ), neo-κ-carrahexaose (κ-κ-κ), and neo-κ-carraoctaose (κ-κ-κ-κ) standards (**Figure 5A**). Together with the sequence based phylogenetical analysis, this experimentally confirmed that Ph1664 is a GH16 endo-active κ-carrageenase. The ability of Ph1664 to degrade both substrates is in accordance with another study showing that κ-carrageenases are capable of cleaving the κ-carrabiose motifs in furcellaran, releasing oligosaccharides of mixed κ- and β-carrabiose nature (Correc et al., 2012). These hybrid products migrate differently to pure κ-motive containing oligosaccharides, to a position between the κ-κ, κ-κ-κ, and κ-κ-κ-κ standards in the FACE. Likewise, the major bands produced by furcellaran hydrolysis, using the cell lysates containing Ph1656 and Ph1663, migrated to positions between κ-carrabiose containing standards and most probably correspond to hybrid κ/β-oligosaccharides. Having observed activity on the polymeric furcellaran, the carrageenolytic PUL-like cluster was re-inspected for other GH candidates likely to possess a similar functional role. Immediately adjacent to the Ph1656 ‘β-carrageenase’ a putative GH42-like β-galactosidase (Ph1657_GH42-like) displaying low sequence identity (18%) to its closest characterized homolog was identified (**Table 1**). In a functional screening against the polymeric substrates (furcellaran and κ-carrageenan) recombinant Ph1657_GH42-like was inactive, but when tested on a commercial β-κ tetrasaccharide, this substrate was indeed hydrolyzed into (β) and (κ) (**Figure 5B**). At first, no activity was observed on furcellaran polysaccharide for Ph1675_GH16, another of the novel ‘β-carrageenase’ homologs. However, when testing this enzyme on the commercial β-κ tetrasaccharide, it was found to degrade the substrate into the corresponding β- and κ-carrabiose units (**Figure 5C**). In addition, Ph1675_GH16 was also capable of further degrading the furcellaran-oligosaccharides produced by Ph1656_GH16 (**Figure 5D**). This suggested that both Ph1657_GH42-like and Ph1675_GH16 are specific for hybrid furcellaran oligosaccharides. An interesting aspect to note here is the fact that this GH16 enzyme, in contrast to most GH16 enzymes, displays more activity against oligosaccharides than on a polymer, suggesting that it might be exo-lytic instead of endo-lytic.

Apart from the recently discovered GH127 α-1,3-(3,6-anhydro)-D-galactosidases (Ficko-Blean et al., 2017), the literature is scarce on glycosidases targeting non-sulfated β-carrageenan motifs, and to the best of our knowledge, only one other study exists. Decades ago (McLean and Williamson, 1981) reported the activity of an enzyme denoted “neocarratetraose 4-*O*-monosulphate β-hydrolase,” which was found to hydrolyze (β-κ) into the resulting (β) and (κ) disaccharides similar to Ph1675_GH16 and Ph1657_GH42-like. However, it remains unclear which of these two enzymes from our current study that corresponds to the activity observed by McLean and Williamson (1981), since this early description of activity was not associated with any sequence information.

Complete hydrolysis of carrageenan into monomers results in D-AHG and D-galactose. Two studies have characterized enzymes responsible for D-AHG conversion. In both *P. atlantica* (Lee et al., 2016) and *Z. galactanivorans* (Ficko-Blean et al., 2017) this pathway consists of a four-step enzymatic reaction resulting in the formation of D-glyceraldehyde-3-phosphate and pyruvate. Homologs to these four enzymes were found in S66^T^ and displayed between 44 and 87% sequence identities to the characterized counterparts from *P. atlantica*. In addition, conserved gene synteny was observed for the D-AHG pathway in all three organisms (Supplementary Figure 3B). Moreover, four sulfatases belonging to the S1_19 sulfatase subfamily were identified interspersed between the GHs. This subfamily contains characterized members (Patl_0889 and Patl_0895 from *P. atlantica* T6c) which cleave off the sulfate from galactose-4-sulfate in ι- and κ-carrageenans, respectively (Prechoux et al., 2013, 2016). Patl_0889 shared 76% identity with the Ph1648 sulfatase from S66^T^ and Patl_0895 showed 83% to Ph1677. Both Ph1648 and Ph1677 sulfatases were located in the carrageenolytic part of the PUL-like cluster. Also, sulfatase genes located in the PUL-like cluster were in general induced with the increased degree of sulfatation in the substrates tested (Supplementary Figure 2C). Altogether, this supported a function in the removal of sulfate groups from the backbone of carrageenan, agar and/or porphyran as reported elsewhere (Kim et al., 2004; Prechoux et al., 2013, 2016; Wang et al., 2015).

**FIGURE 6:**
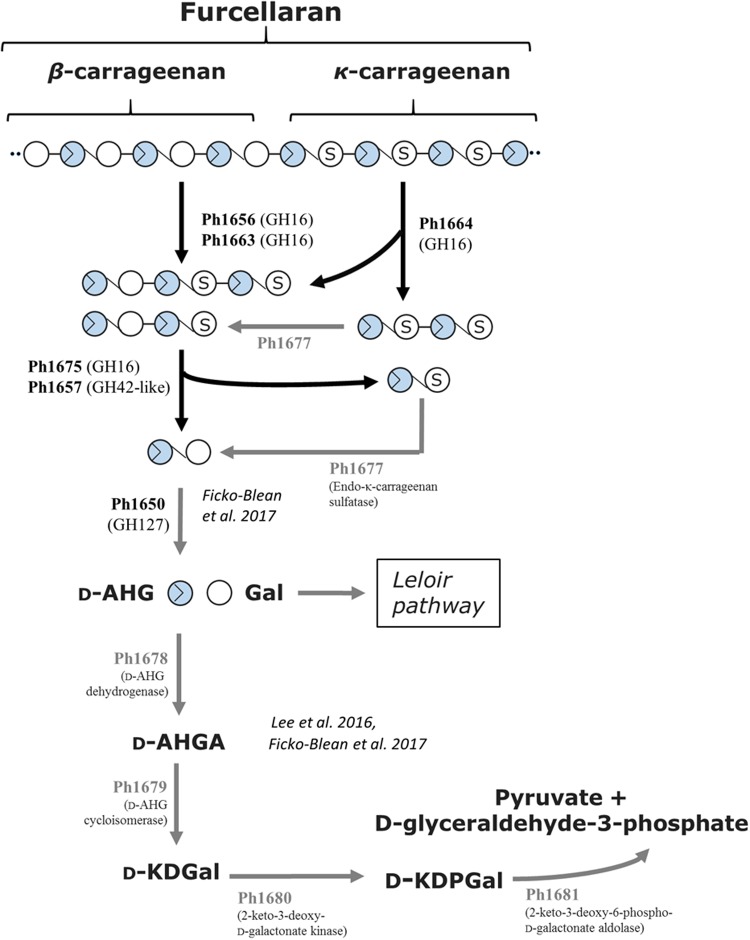
Tentative pathway for carrageenan degradation in *P. hydrolytica* S66^T^. Enzymes are indicated at each reaction step together with CAZy family if applicable. Solid arrows represent the proposed pathway of *P. hydrolytica* S66^T^ based on functional (black) and bioinformatics (gray) analyses. D-AHG, 3,6-anhydro-D-galactose; Gal, D-Galactose; D-AHGA, 3,6-anhydro-D-galactonate; D-KDGal, 2-keto-3-deoxy-D-galactonate; D-KDPGal, 2-keto-3-deoxy-6-phospho-D-galactonate.

On the basis of the current discoveries, we are able for the first time to propose a model for furcellaran degradation in a marine bacterium (**Figure 6**). In S66^T^, it is assumed that the GHs in the carrageenolytic PUL-like cluster each are specific for a particular part in furcellaran. The κ-carrageenase (Ph1664_GH16) targets the κ-carrageenan part in furcellaran releasing κ-carrageenan oligosaccharides that are further desulfated through the action of sulfatases. The proposed sulfatase activity remains to be established. However, a good candidate would be Ph1677, which displays high sequence identity (83%) to a characterized endo-κ-carrageenan sulfatase (Prechoux et al., 2016). The proposed reaction product of the action of the κ-carrageenase Ph1664_GH16 together with sulfatase Ph1677 is mono-sulfated neocarratetraose and neocarrabiose. In addition, enzymes such as the here described furcellaranases, Ph1656_GH16 and Ph1663_GH16, can hydrolyze the β-carrageenan or hybrid part of furcellaran, releasing partially sulfated carrageenan oligosaccharides, e.g., mono-sulfated neocarratetraose. Further degradation of these oligosaccharides is carried out by Ph1675_GH16 and Ph1657_GH42-like enzymes, resulting in neocarrabiose and sulfated neocarrabiose. The sulfated neocarrabiose is further desulfated by Ph1677 as described above, resulting in desulfated neocarrabiose. Finally, the Ph1650_GH127 α-1,3-(3,6-anhydro)-D-galactosidase homolog is proposed to catalyze the reaction step resulting in the formation of D-AHG and galactose (**Figure 6**). Whereas galactose presumably enters directly into the Leloir pathway, D-AHG in S66^T^ is further processed by a D-AHG dehydrogenase (Ph1678) into D-AHGA, subsequently processed by a D-AHG cycloisomerase (Ph1679) into D-KDGal, then by a 2-keto-3-deoxy-D-galactonate kinase (Ph1680) into D-KDPGal and finally by a 2-keto-3-deoxy-6-phospho-D-galactonate aldolase (Ph1681) into D-glyceraldehyde-3-phosphate and pyruvate. The conserved organization of the D-AHG metabolic genes in S66^T^, *P. atlantica* and *Z. galactanivorans* (Supplementary Figure 3B) supports recent data suggesting that this pathway is central in carrageenolytic bacteria (Ficko-Blean et al., 2017).

#### Additional Polysaccharide Utilization Systems of S66^T^

Although strain S66^T^ is able to utilize porphyran as sole carbon source for growth (Bech et al., 2017), genuine porphyranase activity was not detected for the enzymes within the current study. It is possible that depolymerization of porphyran could be carried out by other uncharacterized hydrolases present in the PUL-like clusters. Additionally, the possibility that agarose motifs present in the porphyran substrate are cleaved by agarases and utilized for growth cannot be ruled out. Thus, the route for porphyran degradation in strain S66^T^ remains to be fully uncovered.

In contrast, S66^T^ is unable to utilize ι-carrageenan as sole carbon source (Bech et al., 2017). Yet, a putative ι-carrageenase and ι-carrageenan sulfatase was encoded in the PUL-like cluster in S66^T^. Based on the screening setup used in the current study, it was not possible to validate the function of the putative GH82 ι-carrageenase (Ph1659). Thus, it is unclear whether this gene encodes a genuine ι-carrageenase or, analogous to the discovery of furcellaranases, may target a natural substrate composed of hybrid carrageenan motifs.

### Future Prospects in Enzyme Systems Dedicated to Red Algal Polysaccharide Degradation

It has previously been shown that expression of individual genes in PULs may be regulated by the substrates of the encoded enzymes (D’Elia and Salyers, 1996; Martens et al., 2011; Hehemann et al., 2012; Thomas et al., 2012; Ficko-Blean et al., 2017). For instance, in *Z. galactanivorans*, genes involved in degradation of agarose and porphyran (Hehemann et al., 2012) and κ- and ι-carrageenan (Ficko-Blean et al., 2017) are induced by their polysaccharide substrates. In order to test the regulation of the agarolytic and carrageenolytic parts of the combined PUL-like cluster, S66^T^ was cultivated with various carbon sources (agarose, agar, porphyran or glucose) and the induction pattern of several hydrolytic genes present on the PUL-like cluster was investigated (Supplementary Figure 2). We were unable to obtain data from cells grown with carrageenan, probably due to gel formation on filter membranes used for RNA extraction. More importantly, the formation of cell aggregates during cultivation of S66^T^ on all the substrates prevented optical density comparison of the growth. Instead, this was gauged by the amount of extracted RNA, which was comparable for all treatments (Supplementary Table 4). Consequently, the gene regulation could only be preliminary evaluated.

Overall, our results seem to point toward an even more complex genetic organization of polysaccharide utilizing systems than those reported to date (Ficko-Blean et al., 2017; Grondin et al., 2017). In the here presented case, the co-localization of agar-specific genes with more complex carrageenan degrading genes in the same genetic region, the bi-directional orientation of the genes, and the preliminary gene expression analyses suggest a succession of evolutionary events of gene shuffling and/or horizontal gene transfer, possibly leading to a complex cross-regulation of agarases and carrageenases in S66^T^. Interestingly, this observation is in-line with recent genome wide transcriptomic analyses of *Z. galactanivorans* that show common signatures when growing on chemically divergent polysaccharides from the same algal phylum (Thomas et al., 2017). In our study, both agar-specific and carrageenan-specific genes are regulated by agar and agarose suggesting that the regulation pattern of this PUL-like cluster might be very complex. However, due to the technical limitations in this experiment, we do not yet have access to regulation patterns in the presence of carrageenan and more thorough investigations are needed in order to fully understand the gene regulation in the complex PUL-like cluster in S66^T^. We are currently testing different growth conditions in order to prevent the formation of cell aggregates, which would allow more comprehensive transcriptomic analyses in the future.

The size of the PUL-like region in S66^T^ is unusual and, to our knowledge, one of the biggest reported to date. Thus, the evolutionary events leading to the assembly of this PUL seem intriguing. Although, it was outside the scope of the present study to analyze this in detail, initial observations suggest that many of the genes in the PUL-like region may have been horizontally transferred from other *Gammaproteobacteria*. This is supported by the presence of at least one mobile element within the PUL and DNA/RNA-modifying genes in the flanking regions (not shown).

Functional screening combined with phylogenetic analyses revealed that Ph1656, Ph1663, Ph1675 and possibly Ph1646 constitute a new GH16 subfamily with substrate preference for various stretches of furcellaran – a hybrid carrageenan composed of κ-type and non-sulfated β-type carrabiose motifs. Likewise, Ph1657 was found to cleave a tetrasaccharide (β-κ) into its resulting disaccharide moieties. The exact functionalities are yet to be investigated in detail, but results from the preliminarily screening point toward a group of enzymes acting on the non-sulfated β-carrageenan motifs (since κ-carrageenan was not degraded).

GH16 family enzymes share a β-jellyroll fold and are believed to have evolved from a common ancestor into several individual subfamilies with enzyme activities targeting both marine and terrestrial substrates (Barbeyron et al., 1998; Michel et al., 2006; Eklöf et al., 2013). Thus, detailed biochemical characterization of the novel GH16 members from *P. hydrolytica* S66^T^ is of special interest and should provide new valuable information about their functional roles in the degradation of red algal polysaccharides, as well as the evolutionary relationship between GH16 family enzymes.

Considering the results presented here, it is interesting to note that the furcellaran active GH16 and GH42-like members are absent in the newly characterized carrageenolytic system of *Z. galactanivorans* (except for a genuine GH16 κ-carrageenase). In brief, the carrageenolytic system of *Z. galactanivorans* is composed of a core PUL comprising the D-AHG metabolizing pathway, several sulfatases and α-1,3-(3,6-anhydro)-D-galactosidases, in addition to other genes (including endolytic carrageenases and transporters) encoded in genomic regions outside the core PUL (Ficko-Blean et al., 2017). On the other hand, Ficko-Blean et al. (2017) showed that GH16 and GH42-like homologs with unknown functions are found in carrageenolytic PULs of other marine bacteria including members of the *Alteromonadaceae* family to which *P. hydrolytica* S66^T^ belongs. We therefore speculate that the furcellaranase activities demonstrated in the present study may correspond to these uncharacterized CAZymes. If so, our findings may contribute to a better understanding of carrageenan degradation in marine bacteria.

In addition to the GHs described in this study, other new enzyme activities may be found in the red algal PUL-like clusters. In this context, a GH29 (fucosidases) and a GH63 (glucosidases) located in the agarolytic part of the gene cluster are interesting targets for future characterization studies, since members of these CAZyme families are not obviously involved in hydrolysis of red algal polysaccharides and may therefore represent new enzyme activities. This rationale has earlier led to the discovery of novel enzyme activities acting on algal carbohydrates (Rebuffet et al., 2011; Ficko-Blean et al., 2017). Thus, we expect that future research in other protein complements in the S66^T^ PUL-like clusters may reveal additional functional novelties related to agar and carrageenan degradation.

## Author Contributions

MS-J, PB, MG, TB, MC, and PS conceptualized and designed the studies. MS-J, PB, and TB performed the bioinformatic and phylogenetic analyses. PB and MS-J cloned and characterized the enzymes. RH and PB performed the RT-PCR analyses. MS-J wrote the manuscript with contributions from all authors. All the authors read and approved the final manuscript.

## Conflict of Interest Statement

The authors declare that the research was conducted in the absence of any commercial or financial relationships that could be construed as a potential conflict of interest.

## Footnotes

1 http://rast.nmpdr.org

2 http://abims.sb-roscoff.fr/sulfatlas/

3 http://www.genoscope.cns.fr/agc/mage

## References

[B1] Aarstad O. A. Tondervik A. Sletta H. Skjak-Braek G. (2012). Alginate sequencing: an analysis of block distribution in alginates using specific alginate degrading enzymes. *Biomacromolecules* 13 106–116. 10.1021/bm2013026 22148348

[B2] Anderson N. S. Rees D. A. (1965). Porphyran - a polysaccharide with a masked repeating structure. *J. Chem. Soc.* 5880–5887. 10.1039/jr9650005880

[B3] Arnosti C. (2011). Microbial extracellular enzymes and the marine carbon cycle. *Ann. Rev. Mar. Sci.* 3 401–425. 10.1146/annurev-marine-120709-142731 21329211

[B4] Azam F. Malfatti F. (2007). Microbial structuring of marine ecosystems. *Nat. Rev. Microbiol.* 5 782–791. 10.1038/nrmicro1747 17853906

[B5] Aziz R. K. Bartels D. Best A. A. DeJongh M. Disz T. Edwards R. A. (2008). The RAST Server: rapid annotations using subsystems technology. *BMC Genomics* 9:75. 10.1186/1471-2164-9-75 18261238 PMC2265698

[B6] Barbeyron T. Brillet-Gueguen L. Carre W. Carriere C. Caron C. Czjzek M. (2016a). Matching the diversity of sulfated biomolecules: creation of a classification database for sulfatases reflecting their substrate specificity. *PLoS One* 11:e0164846. 10.1371/journal.pone.0164846 27749924 PMC5066984

[B7] Barbeyron T. Gerard A. Potin P. Henrissat B. Kloareg B. (1998). The kappa-carrageenase of the marine bacterium *Cytophaga drobachiensis*. Structural and phylogenetic relationships within family-16 glycoside hydrolases. *Mol. Biol. Evol.* 15 528–537. 9580981 10.1093/oxfordjournals.molbev.a025952

[B8] Barbeyron T. Thomas F. Barbe V. Teeling H. Schenowitz C. Dossat C. (2016b). Habitat and taxon as driving forces of carbohydrate catabolism in marine heterotrophic bacteria: example of the model algae-associated bacterium *Zobellia galactanivorans* Dsij^T^. *Environ. Microbiol.* 18 4610–4627. 10.1111/1462-2920.13584 27768819

[B9] Bauer M. Kube M. Teeling H. Richter M. Lombardot T. Allers E. (2006). Whole genome analysis of the marine *Bacteroidetes ’Gramella forsetii’* reveals adaptations to degradation of polymeric organic matter. *Environ. Microbiol.* 8 2201–2213. 10.1111/j.1462-2920.2006.01152.x 17107561

[B10] Bech P. K. Schultz-Johansen M. Glaring M. A. Barbeyron T. Czjzek M. Stougaard P. (2017). *Paraglaciecola hydrolytica* sp. nov., a bacterium with hydrolytic activity against multiple seaweed-derived polysaccharides. *Int. J. Syst. Evol. Microbiol.* 67 2242–2247. 10.1099/ijsem.0.001933 28671532

[B11] Blanvillain S. Meyer D. Boulanger A. Lautier M. Guynet C. Denance N. (2007). Plant carbohydrate scavenging through tonB-dependent receptors: a feature shared by phytopathogenic and aquatic bacteria. *PLoS One* 2:e224. 10.1371/journal.pone.0000224 17311090 PMC1790865

[B12] Campo V. L. Kawano D. F. da Silva D. B. Carvalho I. (2009). Carrageenans: biological properties, chemical modifications and structural analysis - A review. *Carbohydr. Polym.* 77 167–180. 10.1016/j.carbpol.2009.01.020

[B13] Chi W. J. Park J. S. Kwak M. J. Kim J. F. Chang Y. K. Hong S. K. (2013). Isolation and characterization of a novel agar-degrading marine bacterium, *Gayadomonas joobiniege* gen, nov, sp. nov., from the Southern Sea, Korea. *J. Microbiol. Biotechnol.* 23 1509–1518. 23966020 10.4014/jmb.1308.08007

[B14] Correc G. Barabanova A. Tuvikene R. Truus K. Yermak I. Helbert W. (2012). Comparison of the structures of hybrid kappa-/beta-carrageenans extracted from *Furcellaria lumbricalis* and *Tichocarpus crinitus*. *Carbohydr. Polym.* 88 31–36. 10.1016/j.carbpol.2011.11.052

[B15] D’Elia J. N. Salyers A. A. (1996). Effect of regulatory protein levels on utilization of starch by *Bacteroides thetaiotaomicron*. *J. Bacteriol.* 178 7180–7186. 8955400 10.1128/jb.178.24.7180-7186.1996 PMC178631

[B16] Eklöf J. M. Shojania S. Okon M. McIntosh L. P. Brumer H. (2013). Structure-function analysis of a broad specificity *Populus trichocarpa* endo-beta-glucanase reveals an evolutionary link between bacterial licheninases and plant XTH gene products. *J. Biol. Chem.* 288 15786–15799. 10.1074/jbc.M113.462887 23572521 PMC3668736

[B17] Enoki T. Tominaga T. Takashima F. Ohnogi H. Sagawa H. Kato I. (2012). Anti-tumor-promoting activities of agaro-oligosaccharides on two-stage mouse skin carcinogenesis. *Biol. Pharm. Bull.* 35 1145–1149. 10.1248/bpb.b12-00188 22791164

[B18] Ficko-Blean E. Prechoux A. Thomas F. Rochat T. Larocque R. Zhu Y. (2017). Carrageenan catabolism is encoded by a complex regulon in marine heterotrophic bacteria. *Nat. Commun.* 8:1685. 10.1038/s41467-017-01832-6 29162826 PMC5698469

[B19] Finn R. D. Coggill P. Eberhardt R. Y. Eddy S. R. Mistry J. Mitchell A. L. (2016). The Pfam protein families database: towards a more sustainable future. *Nucleic Acids Res.* 44 D279–D285. 10.1093/nar/gkv1344 26673716 PMC4702930

[B20] Foran E. Buravenkov V. Kopel M. Mizrahi N. Shoshani S. Helbert W. (2017). Functional characterization of a novel “ulvan utilization loci” found in *Alteromonas* sp LOR genome. *Algal Res.* 25 39–46. 10.1016/j.algal.2017.04.036

[B21] Gao B. Jin M. Li L. Qu W. Zeng R. (2017). Genome sequencing reveals the complex polysaccharide-degrading ability of novel deep-sea bacterium *Flammeovirga pacifica* WPAGA1. *Front. Microbiol.* 8:600. 10.3389/fmicb.2017.00600 28443080 PMC5385347

[B22] Geu-Flores F. Nour-Eldin H. H. Nielsen M. T. Halkier B. A. (2007). USER fusion: a rapid and efficient method for simultaneous fusion and cloning of multiple PCR products. *Nucleic Acids Res.* 35:e55. 10.1093/nar/gkm106 17389646 PMC1874642

[B23] Grondin J. M. Tamura K. Dejean G. Abbott D. W. Brumer H. (2017). Polysaccharide utilization loci: fueling microbial communities. *J. Bacteriol.* 199:e00860-16. 10.1128/JB.00860-16 28138099 PMC5512228

[B24] Hehemann J. H. Correc G. Barbeyron T. Helbert W. Czjzek M. Michel G. (2010). Transfer of carbohydrate-active enzymes from marine bacteria to Japanese gut microbiota. *Nature* 464 908–912. 10.1038/nature08937 20376150

[B25] Hehemann J. H. Correc G. Thomas F. Bernard T. Barbeyron T. Jam M. (2012). Biochemical and structural characterization of the complex agarolytic enzyme system from the marine bacterium *Zobellia galactanivorans*. *J. Biol. Chem.* 287 30571–30584. 10.1074/jbc.M112.377184 22778272 PMC3436304

[B26] Helbert W. (2017). Marine polysaccharide sulfatases. *Front. Mar. Sci.* 4:6 10.3389/fmars.2017.00006

[B27] Hu B. Gong Q. Wang Y. Ma Y. Li J. Yu W. (2006). Prebiotic effects of neoagaro-oligosaccharides prepared by enzymatic hydrolysis of agarose. *Anaerobe* 12 260–266. 10.1016/j.anaerobe.2006.07.005 16973391

[B28] Juncker A. S. Willenbrock H. Von Heijne G. Brunak S. Nielsen H. Krogh A. (2003). Prediction of lipoprotein signal peptides in Gram-negative bacteria. *Protein Sci.* 12 1652–1662. 10.1110/ps.0303703 12876315 PMC2323952

[B29] Kabisch A. Otto A. Konig S. Becher D. Albrecht D. Schuler M. (2014). Functional characterization of polysaccharide utilization loci in the marine *Bacteroidetes ’Gramella forsetii’*, KT0803. *ISME J.* 8 1492–1502. 10.1038/ismej.2014.4 24522261 PMC4069401

[B30] Kalitnik A. A. Anastyuk S. D. Sokolova E. V. Kravchenko A. O. Khasina E. I. Yermak I. M. (2016). Oligosaccharides of kappa/beta-carrageenan from the red alga *Tichocarpus crinitus* and their ability to induce interleukin 10. *J. Appl. Phycol.* 28 545–553. 10.1007/s10811-015-0577-6

[B31] Kim J. H. Byun D. S. Godber J. S. Choi J. S. Choi W. C. Kim H. R. (2004). Purification and characterization of arylsulfatase from *Sphingomonas* sp AS6330. *Appl. Microbiol. Biotechnol.* 63 553–559. 10.1007/s00253-003-1463-8 14600791

[B32] Kim S. G. Pheng S. Lee Y. J. Eom M. K. Shin D. H. (2016). *Agarivorans aestuarii* sp nov., an agar-degrading bacterium isolated from a tidal flat. *Int. J. Syst. Evol. Microbiol.* 66 3119–3124. 10.1099/ijsem.0.001155 27189058

[B33] Knutsen S. H. Myslabodski D. E. Grasdalen H. (1990). Characterization of carrageenan fractions from Norwegian *Furcellaria-lumbricalis* (Huds) Lamour by H-1-Nmr spectroscopy. *Carbohydr. Res.* 206 367–372. 10.1016/0008-6215(90)80076-f

[B34] Knutsen S. H. Myslabodski D. E. Larsen B. Usov A. I. (1994). A modified system of nomenclature for red algal galactans. *Bot. Mar.* 37 163–169. 10.1515/botm.1994.37.2.163

[B35] Lee C. H. Kim H. T. Yun E. J. Lee A. R. Kim S. R. Kim J. H. (2014). A novel agarolytic beta-galactosidase acts on agarooligosaccharides for complete hydrolysis of agarose into monomers. *Appl. Environ. Microbiol.* 80 5965–5973. 10.1128/AEM.01577-14 25038102 PMC4178691

[B36] Lee S. B. Cho S. J. Kim J. A. Lee S. Y. Kim S. M. Lim H. S. (2014). Metabolic pathway of 3,6-anhydro-L-galactose in agar-degrading microorganisms. *Biotechnol. Bioprocess Eng.* 19 866–878. 10.1007/s12257-014-0622-3

[B37] Lee S. B. Kim J. A. Lim H. S. (2016). Metabolic pathway of 3,6-anhydro-D-galactose in carrageenan-degrading microorganisms. *Appl. Microbiol. Biotechnol.* 100 4109–4121. 10.1007/s00253-016-7346-6 26875872

[B38] Lombard V. Golaconda Ramulu H. Drula E. Coutinho P. M. Henrissat B. (2014). The carbohydrate-active enzymes database (CAZy) in 2013. *Nucleic Acids Res.* 42 D490–D495. 10.1093/nar/gkt1178 24270786 PMC3965031

[B39] Mann A. J. Hahnke R. L. Huang S. Werner J. Xing P. Barbeyron T. (2013). The genome of the alga-associated marine flavobacterium *Formosa agariphila* KMM 3901T reveals a broad potential for degradation of algal polysaccharides. *Appl. Environ. Microbiol.* 79 6813–6822. 10.1128/AEM.01937-13 23995932 PMC3811500

[B40] Martens E. C. Koropatkin N. M. Smith T. J. Gordon J. I. (2009). Complex glycan catabolism by the human gut microbiota: the *Bacteroidetes* Sus-like paradigm. *J. Biol. Chem.* 284 24673–24677. 10.1074/jbc.R109.022848 19553672 PMC2757170

[B41] Martens E. C. Lowe E. C. Chiang H. Pudlo N. A. Wu M. McNulty N. P. (2011). Recognition and degradation of plant cell wall polysaccharides by two human gut symbionts. *PLoS Biol.* 9:e1001221. 10.1371/journal.pbio.1001221 22205877 PMC3243724

[B42] Martin M. Barbeyron T. Martin R. Portetelle D. Michel G. Vandenbol M. (2015). The cultivable surface microbiota of the brown alga *Ascophyllum nodosum* is Enriched in Macroalgal-polysaccharide-degrading bacteria. *Front. Microbiol.* 6:1487. 10.3389/fmicb.2015.01487 26734000 PMC4690005

[B43] Martin M. Portetelle D. Michel G. Vandenbol M. (2014). Microorganisms living on macroalgae: diversity, interactions, and biotechnological applications. *Appl. Microbiol. Biotechnol.* 98 2917–2935. 10.1007/s00253-014-5557-2 24562178

[B44] McLean M. W. Williamson F. B. (1981). Neocarratetraose 4-O-monosulphate beta-hydrolase from *Pseudomonas carrageenovora*. *Eur. J. Biochem.* 113 447–456. 7215336 10.1111/j.1432-1033.1981.tb05084.x

[B45] Michel G. Nyval-Collen P. Barbeyron T. Czjzek M. Helbert W. (2006). Bioconversion of red seaweed galactans: a focus on bacterial agarases and carrageenases. *Appl. Microbiol. Biotechnol.* 71 23–33. 10.1007/s00253-006-0377-7 16550377

[B46] Morya V. K. Kim J. Kim E. K. (2012). Algal fucoidan: structural and size-dependent bioactivities and their perspectives. *Appl. Microbiol. Biotechnol.* 93 71–82. 10.1007/s00253-011-3666-8 22089385

[B47] Neumann A. M. Balmonte J. P. Berger M. Giebel H. A. Arnosti C. Voget S. (2015). Different utilization of alginate and other algal polysaccharides by marine *Alteromonas macleodii* ecotypes. *Environ. Microbiol.* 17 3857–3868. 10.1111/1462-2920.12862 25847866

[B48] Nørholm M. H. (2010). A mutant Pfu DNA polymerase designed for advanced uracil-excision DNA engineering. *BMC Biotechnol.* 10:21. 10.1186/1472-6750-10-21 20233396 PMC2847956

[B49] Nour-Eldin H. H. Geu-Flores F. Halkier B. A. (2010). USER cloning and USER fusion: the ideal cloning techniques for small and big laboratories. *Methods Mol. Biol.* 643 185–200. 10.1007/978-1-60761-723-5_13 20552452

[B50] Petersen T. N. Brunak S. von Heijne G. Nielsen H. (2011). SignalP 4.0: discriminating signal peptides from transmembrane regions. *Nat. Methods* 8 785–786. 10.1038/nmeth.1701 21959131

[B51] Prechoux A. Genicot S. Rogniaux H. Helbert W. (2013). Controlling carrageenan structure using a novel formylglycine-dependent sulfatase, an endo-4S-iota-carrageenan sulfatase. *Mar. Biotechnol.* 15 265–274. 10.1007/s10126-012-9483-y 23011004

[B52] Prechoux A. Genicot S. Rogniaux H. Helbert W. (2016). Enzyme-assisted preparation of furcellaran-like kappa-/beta-Carrageenan. *Mar. Biotechnol.* 18 133–143. 10.1007/s10126-015-9675-3 26585588

[B53] Rebuffet E. Groisillier A. Thompson A. Jeudy A. Barbeyron T. Czjzek M. (2011). Discovery and structural characterization of a novel glycosidase family of marine origin. *Environ. Microbiol.* 13 1253–1270. 10.1111/j.1462-2920.2011.02426.x 21332624

[B54] Schultz-Johansen M. Glaring M. A. Bech P. K. Stougaard P. (2016). Draft genome sequence of a novel marine bacterium, *Paraglaciecola* sp. strain S66, with hydrolytic activity against seaweed polysaccharides. *Genome Announc.* 4:e00304-16. 10.1128/genomeA.00304-16 27103729 PMC4841144

[B55] Starr C. M. Masada R. I. Hague C. Skop E. Klock J. C. (1996). Fluorophore-assisted carbohydrate electrophoresis in the separation, analysis, and sequencing of carbohydrates. *J. Chromatogr. A* 720 295–321. 8601197 10.1016/0021-9673(95)00749-0

[B56] Studier F. W. (2005). Protein production by auto-induction in high density shaking cultures. *Protein Expr. Purif.* 41 207–234. 15915565 10.1016/j.pep.2005.01.016

[B57] Sun C. Fu G. Y. Zhang C. Y. Hu J. Xu L. Wang R. J. (2016). Isolation and complete genome sequence of *Algibacter alginolytica* sp. nov., a novel seaweed-degrading *Bacteroidetes* bacterium with diverse putative polysaccharide utilization loci. *Appl. Environ. Microbiol.* 82 2975–2987. 10.1128/AEM.00204-16 26969704 PMC4959061

[B58] Tamura K. Stecher G. Peterson D. Filipski A. Kumar S. (2013). MEGA6: molecular evolutionary genetics analysis version 6.0. *Mol. Biol. Evol.* 30 2725–2729. 10.1093/molbev/mst197 24132122 PMC3840312

[B59] Teeling H. Fuchs B. M. Becher D. Klockow C. Gardebrecht A. Bennke C. M. (2012). Substrate-controlled succession of marine bacterioplankton populations induced by a phytoplankton bloom. *Science* 336 608–611. 10.1126/science.1218344 22556258

[B60] Teeling H. Fuchs B. M. Bennke C. M. Kruger K. Chafee M. Kappelmann L. (2016). Recurring patterns in bacterioplankton dynamics during coastal spring algae blooms. *Elife* 5:e11888. 10.7554/eLife.11888 27054497 PMC4829426

[B61] Terrapon N. Henrissat B. (2014). How do gut microbes break down dietary fiber? *Trends Biochem. Sci.* 39 156–158. 10.1016/j.tibs.2014.02.005 24613530

[B62] Thomas F. Barbeyron T. Michel G. (2011). Evaluation of reference genes for real-time quantitative PCR in the marine flavobacterium *Zobellia galactanivorans*. *J. Microbiol. Methods* 84 61–66. 10.1016/j.mimet.2010.10.016 21047531

[B63] Thomas F. Barbeyron T. Tonon T. Genicot S. Czjzek M. Michel G. (2012). Characterization of the first alginolytic operons in a marine bacterium: from their emergence in marine *Flavobacteriia* to their independent transfers to marine *Proteobacteria* and human gut *Bacteroides*. *Environ. Microbiol.* 14 2379–2394. 10.1111/j.1462-2920.2012.02751.x 22513138

[B64] Thomas F. Bordron P. Eveillard D. Michel G. (2017). Gene expression analysis of *Zobellia galactanivorans* during the degradation of algal polysaccharides reveals both substrate-specific and shared transcriptome-wide responses. *Front. Microbiol.* 8:1808. 10.3389/fmicb.2017.01808 28983288 PMC5613140

[B65] Usov A. I. (2011). Polysaccharides of the red algae. *Adv. Carbohydr. Chem. Biochem.* 65 115–217. 10.1016/B978-0-12-385520-6.00004-2 21763512

[B66] Vallenet D. Engelen S. Mornico D. Cruveiller S. Fleury L. Lajus A. (2009). MicroScope: a platform for microbial genome annotation and comparative genomics. *Database* 2009:ba021. 10.1093/database/bap021 20157493 PMC2790312

[B67] von Scheibner M. Sommer U. Jurgens K. (2017). Tight coupling of *Glaciecola* spp. and diatoms during cold-water phytoplankton spring blooms. *Front. Microbiol.* 8:27. 10.3389/fmicb.2017.00027 28154558 PMC5243806

[B68] Wang X. Y. Duan D. L. Xu J. C. Gao X. Fu X. T. (2015). Characterization of a novel alkaline arylsulfatase from *Marinomonas* sp FW-1 and its application in the desulfation of red seaweed agar. *J. Ind. Microbiol. Biotechnol.* 42 1353–1362. 10.1007/s10295-015-1625-6 26286088

[B69] Wietz M. Wemheuer B. Simon H. Giebel H. A. Seibt M. A. Daniel R. (2015). Bacterial community dynamics during polysaccharide degradation at contrasting sites in the Southern and Atlantic Oceans. *Environ. Microbiol.* 17 3822–3831. 10.1111/1462-2920.12842 25753990

[B70] Xing P. Hahnke R. L. Unfried F. Markert S. Huang S. Barbeyron T. (2015). Niches of two polysaccharide-degrading *Polaribacter* isolates from the North Sea during a spring diatom bloom. *ISME J.* 9 1410–1422. 10.1038/ismej.2014.225 25478683 PMC4438327

[B71] Yan S. L. Yu M. Wang Y. Shen C. Zhang X. H. (2011). *Catenovulum agarivorans* gen. nov., sp nov., a peritrichously flagellated, chain-forming, agar-hydrolysing gammaproteobacterium from seawater. *Int. J. Syst. Evol. Microbiol.* 61 2866–2873. 10.1099/ijs.0.027565-0 21257691

[B72] Yang B. Yu G. L. Zhao X. Ren W. N. Jiao G. L. Fang L. H. (2011). Structural characterisation and bioactivities of hybrid carrageenan-like sulphated galactan from red alga *Furcellaria lumbricalis*. *Food Chem.* 124 50–57. 10.1016/j.foodchem.2010.05.102

[B73] Yin Y. Mao X. Yang J. Chen X. Mao F. Xu Y. (2012). dbCAN: a web resource for automated carbohydrate-active enzyme annotation. *Nucleic Acids Res.* 40 W445–W451. 10.1093/nar/gks479 22645317 PMC3394287

[B74] Yong J. J. Park S. J. Kim H. J. Rhee S. K. (2007). *Glaciecola agarilytica* sp. nov., an agar-digesting marine bacterium from the East Sea, Korea. *Int. J. Syst. Evol. Microbiol.* 57(Pt 5), 951–953. 10.1099/ijs.0.64723-0 17473239

[B75] Yun E. J. Lee S. Kim H. T. Pelton J. G. Kim S. Ko H. J. (2015). The novel catabolic pathway of 3,6-anhydro-L-galactose, the main component of red macroalgae, in a marine bacterium. *Environ. Microbiol.* 17 1677–1688. 10.1111/1462-2920.12607 25156229

[B76] Zhu Y. Chen P. Bao Y. Men Y. Zeng Y. Yang J. (2016). Complete genome sequence and transcriptomic analysis of a novel marine strain *Bacillus weihaiensis* reveals the mechanism of brown algae degradation. *Sci. Rep.* 6:38248. 10.1038/srep38248 27901120 PMC5128808

