# Supplementary material for: A Novel Enzyme Portfolio for Red Algal Polysaccharide Degradation in the Marine Bacterium Paraglaciecola hydrolytica S66T Encoded in a Sizeable Polysaccharide Utilization Locus
Source: Front Microbiol. 2018 May 3;9:839. doi: 10.3389/fmicb.2018.00839 (PMC5943477; doi:10.3389/fmicb.2018.00839)
Supplement: Supplementary file 1 [file Presentation_1.pdf]

Supplementary Material

A novel enzyme portfolio for red algal polysaccharide degradation in the marine bacterium *Paraglaciecola hydrolytica* S66^T encoded in a sizeable polysaccharide utilization locus

Mikkel Schultz-Johansen[1], Pernille Kjersgaard Bech[1], Rosanna Catherine Hennessy[1], Mikkel Andreas Glaring[1], Tristan Barbeyron[2], Mirjam Czjzek[2] and Peter Stougaard[1*]

[1]Section for Microbial Ecology and Biotechnology, Department of Plant and Environmental Sciences, University of Copenhagen, Frederiksberg, Denmark

[2]Sorbonne Université, CNRS, Integrative Biology of Marine Models (LBI2M), Station Biologique de Roscoff (SBR), 29680 Roscoff, France

*** Correspondence:** psg@plen.ku.dk

Supplementary Table 1. Primers and oligonucleotides used in this study. Overhangs designed for USER^{TM} cloning are underlined. Restriction sites are marked in italics.

Gene construct	Forward primer sequence (5'-3')	Reverse primer sequence (5'-3')	Cloning strategy	Nucleotides
Ph1566	ATATGGCUCAAACTAATGGGCC	ACTTCCACUAGTTCTATTAAGTGTGAATG	pET9a-USER-2	67-2560
Ph1586	GGCTTAAUGCGTCGGTGAAG	GGTTTAAUTGCATCACAAGGAGCAC	pET9a-USER-1	67-2274
Ph1589	GGCTTAAUGCAGATAAACAGGCC	GGTTTAAUTTTATTGTCGTTGTATCTGCGT	pET9a-USER-1	67-2331
Ph1607	GGCTTAAUTGTGGCGGATCG	GGTTTAAUTTCAGCCCTTTTTAAGGCTTTAT	pET9a-USER-1	73-2397
Ph1609	GGCTTAAUGAAAACGTACAATCTCCAG	GGTTTAAUTTGTTCAAAGCGCG	pET9a-USER-1	67-2313
Ph1615	GGCTTAAUATGTCAACAAACAAGATA	GGTTTAAUATGTTTATTCTGGAAGGTAC	pET9a-USER-1	1-1077
Ph1624	GGCTTAAUCGACAAAACAGTGAGC	GGTTTAAUTGGTCCTTGTTCTGCC	pET9a-USER-1	100-2364
Ph1631	ATA*CATATG*ACCATGGCAGATTGGGATGAC	TATA*CTCGAG*TTTGCTGTCTACCGGTTTATA	pET22b (*Nde*I/*Xho*I)	59-879
Ph1636	GGCTTAAUTTTTTACTGATGTCTGGTAACG	GGTTTAAUATTTGAACGGGTATGAGCG	pET9a-USER-1	43-2199
Ph1656	GGCTTAAUGAGGTATTACCTTTATCAGATCA	GGTTTAAUCTTTTGCCATACTCGCA	pET9a-USER-1	178-1074
Ph1657	GGCTTAAUTCTGAGCAAAGTAAACAAG	GGTTTAAUAAGCGCGTTGACCTC	pET9a-USER-1	64-2430
Ph1659	GGCTTAAUGTTGAGCGTCAAATGACCT	GGTTTAAUATCACATGCAAATAGCTTTTCATT	pET9a-USER-1	58-1260
Ph1663	GGCTTAAUATATTGCCATTATCAGATCCAAAA	GGTTTAAUTTCCTCCTGCGGTATTTTACTAA	pET9a-USER-1	73-1389
Ph1664	GGCTTAAUATGTTAAGCCTTAGCGCTTG	GGTTTAAUTTGGCGCGGTTTGTA	pET9a-USER-1	34-966
Ph1675	ATATGGCUAACGTACTGCCTCTTTCG	ACTTCCACUTTCTGGTGTTTGCCACAC	pET9a-USER-2	103-1041
USER cassette	TATGCTGAGGCTTAATTAAACCTCAGCCGCA<br>CCACCACCACCACCACCACCACTAAG	GATCCTTAGTGGTGGTGGTGGTGGTGGTGGT<br>GCGGCTGAGGTTTAATTAAGCCTCAGCA	pET9a (*Nde*I/*Bam*HI)	
pET9a-USER-2	AGTGGAAGUCCGCACCACCACCAC	AGCCATAUGTATATCTCCTTCTTAAAG		

Supplementary Table 2. Primers used in the RT-PCR transcription analysis of genes from *Paraglaciecola hydrolytica* S66^{T}.

**Target gene**	**Forward primer sequence (5'-3')**	**Reverse primer sequence (5'-3')**	**Product length (bp)**
gyrB	CAATCGATGAAGCTCTAGCG	GAATAATGCAAACGCCTGAG	474
Ph1586	GTTATTTCTGAGTACGGCGC	CAAGGAAAGCAAGGTCAACG	463
Ph1589	GCTGGATAACTTGCGCTTAC	GTCAGAGGCAATATAACGGC	472
Ph1596	GGCGCTACAGCTATTGATTG	CCCAGTCCATTTGCGCAATG	496
Ph1607	CGATTACATGGCGCGTTATG	GTTTAGGCGCACCAAAACTG	464
Ph1609	GCCATCGTTGATCAGTATGG	GCTCTTCATAGCTTGGTAAC	458
Ph1615	CATATCGCTATTGCGTGGGC	CTGGAGCCCCTTTTACATGG	463
Ph1617	CAAGTGGCATCAAGGTGATG	GATTCGCTTTAGTGCCACTG	506
Ph1618	GGTGGTCATGGTTATTTTCC	CAATGGGCAATAAAGGGCAC	485
Ph1624	GGTATCGATTATCGCGATCC	GCAAATACTGGATCAAAGGG	495
Ph1629	GATGATCATAAACCGACTCC	CAAAAACTCTGACCATCGAG	458
Ph1631	GCAGATTGGGATGACATTCC	GGCTCACGTACAAAAACATG	476
Ph1636	GTTTGGTGGTTTGTGGATCC	CTAGGATCAAACACATCGTG	449
Ph1646	GGCTATAGGTTACCAGTCTG	GGCGTGGATTTTGTTCAAAC	473
Ph1647	CATGAACATGCCAATGAGCG	GTCGATCATGGCGATAGCAC	450
Ph1648	CGCCGGATATTTTACTGGTG	CACTCATGAACACAATCAGG	506
Ph1656	CTGTTTACCAACACCCAGAC	GCCACCCGAAAACCTAAATC	457
Ph1659	GACATCACTGTCATCAATGC	CAACTCACAAGGTACAAAGG	474
Ph1663	CGAAAATATCACCACAGCCG	CTTTACTGGGAATGCCATGC	455
Ph1664	CGATGATTGGGAATACGTAG	CATGTCCAGCACTTCTGGTC	482
Ph1675	GAGCCCGACTTTCCTTTTAG	GCTTCGCTAAAAAGCTTGCC	467

Supplementary Table 3: Enzymes from *P. hydrolytica* S66^{T} located in a PUL-like gene cluster and with a proposed involvement in the degradation of red algal polysaccharides. Characterized homologs are indicated together with the putative function. *Ph1592/93 each represents part of the 2-keto-3-deoxy-L-galactonate-5-dehydrogenase, but was split in the automatic annotation procedure due to a sequencing gap. Percent identity is reported for the closest hit in the PDB database or alternatively for characterized homologs obtained from the studies of [1](Lee et al., 2014a), [2](Ficko-Blean et al., In Press), [3](Prechoux et al., 2016), [4](Prechoux et al., 2013), [5](Lee et al., 2014b), [6](Lee et al., 2016).

Locus tag	Enzyme family	Predicted function	Accession no. of best characterized hit	Percent identity
Ph1566	GH2	Agarolytic β-galactosidase	AEX22320.1 (*Vibrio* sp. EJY3)	40 % [1]
Ph1567	GH29	α-fucosidase	4NI3_A (*Fusarium graminearum*)	34 %
Ph1570	GH63	Mannosylglycerate hydrolase	4WVA_A (*Thermus thermophilus* HB8)	43 %
Ph1586	GH86	β-Porphyranase	4AW7_A (*Bacteroides Plebeius* M12^{T})	33 %
Ph1589	GH50	Exo-β-agarase	4BQ2_A (*Saccharophagus degradans* 2-40^{T})	46 %
Ph1607	GH86	β-Porphyranase	4AW7_A (*Bacteroides Plebeius* M12^{T})	33 %
Ph1609	GH50	Exo-β-agarase	4BQ2_A (*Saccharophagus degradans* 2-40^{T})	61 %
Ph1615	GH117	α-1,3-(3,6-anhydro)-L-galactosidase	3R4Y_A (*Saccharophagus degradans* 2-40^{T})	73 %
Ph1624	GH50	Exo-β-agarase	4BQ2_A (*Saccharophagus degradans* 2-40^{T})	47 %
Ph1631	GH16	β-agarase	3WZ1_A (*Microbulbifer thermotolerans* JAMB A94^{T})	68 %
Ph1636	GH50	Exo-β-agarase	4BQ2_A (*Saccharophagus degradans* 2-40^{T})	31 %
Ph1646	GH16	β-Porphyranase	4ATE_A (*Zobellia galactanivorans* Dsij^{T})	33 %
Ph1650	GH127	α-1,3-(3,6-anhydro)-D-galactosidase	ZGAL_3148/ZGAL_3150 (*Z. galactanivorans* Dsij^{T})	60/62 % [2]
Ph1656	GH16	β-Porphyranase	3JUU_A (*Zobellia galactanivorans* Dsij^{T})	26 %
Ph1657	GH42-like	β-galactosidase	5DFA _A (*Geobacillus stearothermophilus* T6)	18 %
Ph1659	GH82	ι-carrageenase	1H80_A (*"Alteromonas fortis"* 1)	33 %
Ph1663	GH16	β-Porphyranase	4ATE_A (*Zobellia galactanivorans* Dsij^{T})	29 %
Ph1664	GH16	κ-carrageenase	1DYP_A (*Pseudoalteromonas carrageenovora* 9^{T})	40 %
Ph1675	GH16	β-Porphyranase	4ATE_A (*Zobellia galactanivorans* Dsij^{T})	27 %
Ph1596	S1_15	Sulfatase	1E2S_P (*Homo sapiens*)	27 %
Ph1617	S1_19	Endo-4S-κ-carrageenan sulfatase	ABG39421.1(*Pseudoalteromonas atlantica* T6c)	58 % [3]
Ph1618	S1_19	Sulfatase	1FSU_A (*Homo sapiens*)	35 %
Ph1629	S1_24	Sulfatase	3ED4_A (*Escherischia coli* O6:H1 UPEC)	26 %
Ph1647	S1_N.C.	Sulfatase	4UPH_A (*Rhizobium radiobacter* K84)	29 %
Ph1648	S1_19	Endo-4S-ι-carrageenan sulfatase	ABG39415.1 (*Pseudoalteromonas atlantica* T6c)	76 % [4]
Ph1677	S1_19	Endo-4S-κ-carrageenan sulfatase	ABG39421.1 (*Pseudoalteromonas atlantica* T6c)	83 % [3]
Ph1595	-	L-AHG dehydrogenase	ABG41069.1 (*Pseudoalteromonas atlantica* T6c)	87 % [5]
Ph1591	-	L-AHG cycloisomerase	ABG41066.1 (*Pseudoalteromonas atlantica* T6c)	71 % [5]
Ph1592/93*	-	2-keto-3-deoxy-L-galactonate-5-dehydrogenase	ABG41067.1 (*Pseudoalteromonas atlantica* T6c)	92/89 % [5]
Ph1594	-	2,5-diketo-3-deoxy-L-galactonate-5-dehydrogenase	ABG41068.1 (*Pseudoalteromonas atlantica* T6c)	89 % [5]
Ph1616	-	KDG kinase	ABG40487.1 (*Pseudoalteromonas atlantica* T6c)	49 % [5]
Ph1678	-	D-AHG dehydrogenase	ABG39422.1 (*Pseudoalteromonas atlantica* T6c)	69 % [6]
Ph1679	-	D-AHG cycloisomerase	ABG39423.1 (*Pseudoalteromonas atlantica* T6c)	87 % [6]
Ph1680	-	2-keto-3-deoxy-D-galactonate kinase	ABG39424.1 (*Pseudoalteromonas atlantica* T6c)	44 % [6]
Ph1681	-	2-keto-3-deoxy-6-phospho-D-galactonate aldolase	ABG39425.1 (*Pseudoalteromonas atlantica* T6c)	66 % [6]

Supplementary Table 4: Yield of RNA recovered from S66T cells grown on different carbon sources. DNase treatment was performed on extracted RNA and PCR amplification was used to verify the complete removal of genomic DNA prior to cDNA synthesis.

	**Crude RNA (ng/µl)**			**DNase Treated RNA (ng/µl)**		
**Substrate/Replicate**	**R1**	**R2**	**R3**	**R1**	**R2**	**R3**
**Agarose***	540	560	550	17.9	21.5	14.8
**Agar***	108	313	231	11.8	16.1	19.1
**Porphyran**	283	225	145	240	258	131
**Glucose**	380	353	342	367	299	232

*****Rigorous DNase treatment performed to remove contaminating genomic DNA.

Supplementary Figure 1: Structural representation of repetitive disaccharide motifs (-*bioses*) commonly found in agar **(A)** and carrageenan **(B)**. A “Neo” prefix is used to distinguish motifs with an α-1,3 moiety at the non-reducing end from those with a β-1,4 moiety at the non-reducing end (as exemplified for two of the structural representations).

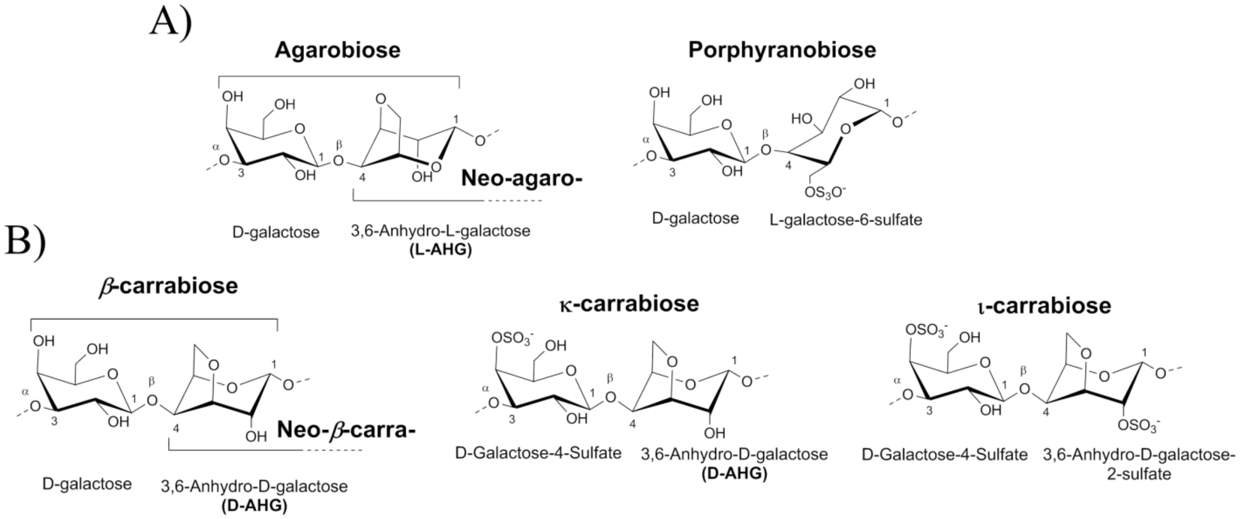

Supplementary Figure 2: RT-PCR transcription analysis of GH genes within the PUL-like cluster. Gene induction was tested for biological replicates of *P. hydrolytica* S66^{T} grown in the presence of agar-type substrates relative to a glucose control. *gyrB* was used as reference gene. **A):** Agarolytic genes, **B):** Carrageenolytic genes, **C):** Sulfatases

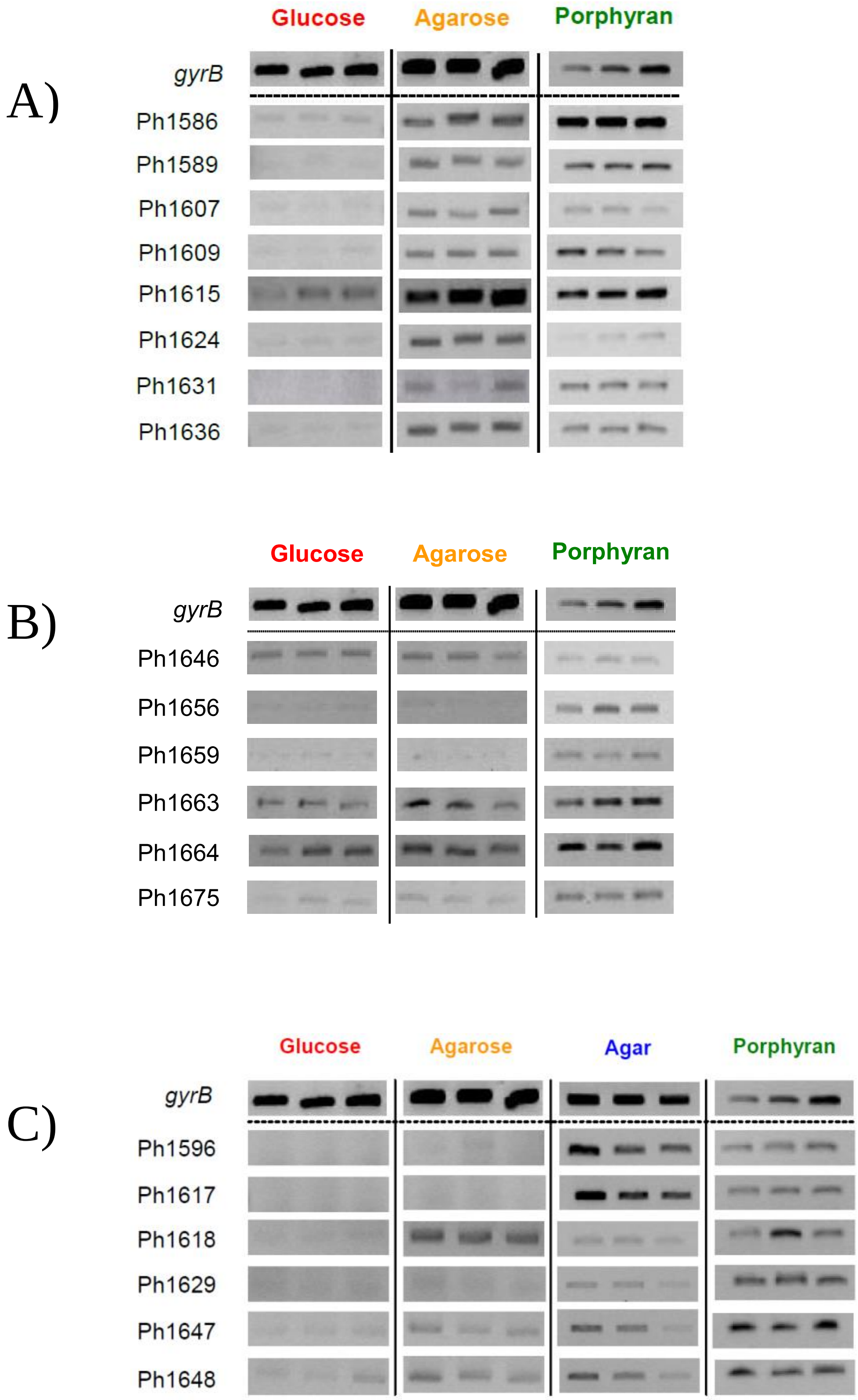

Supplementary Figure 3: Genomic organization of L-AHG and D-AHG metabolizing genes in *P. hydrolytica* S66[T], *Z. galactanivorans and P. atlantica*. Genes are colour-coded according to function and the shared sequence identities (protein) between the organisms are shown. * indicates that gene functions have been experimentally verified.

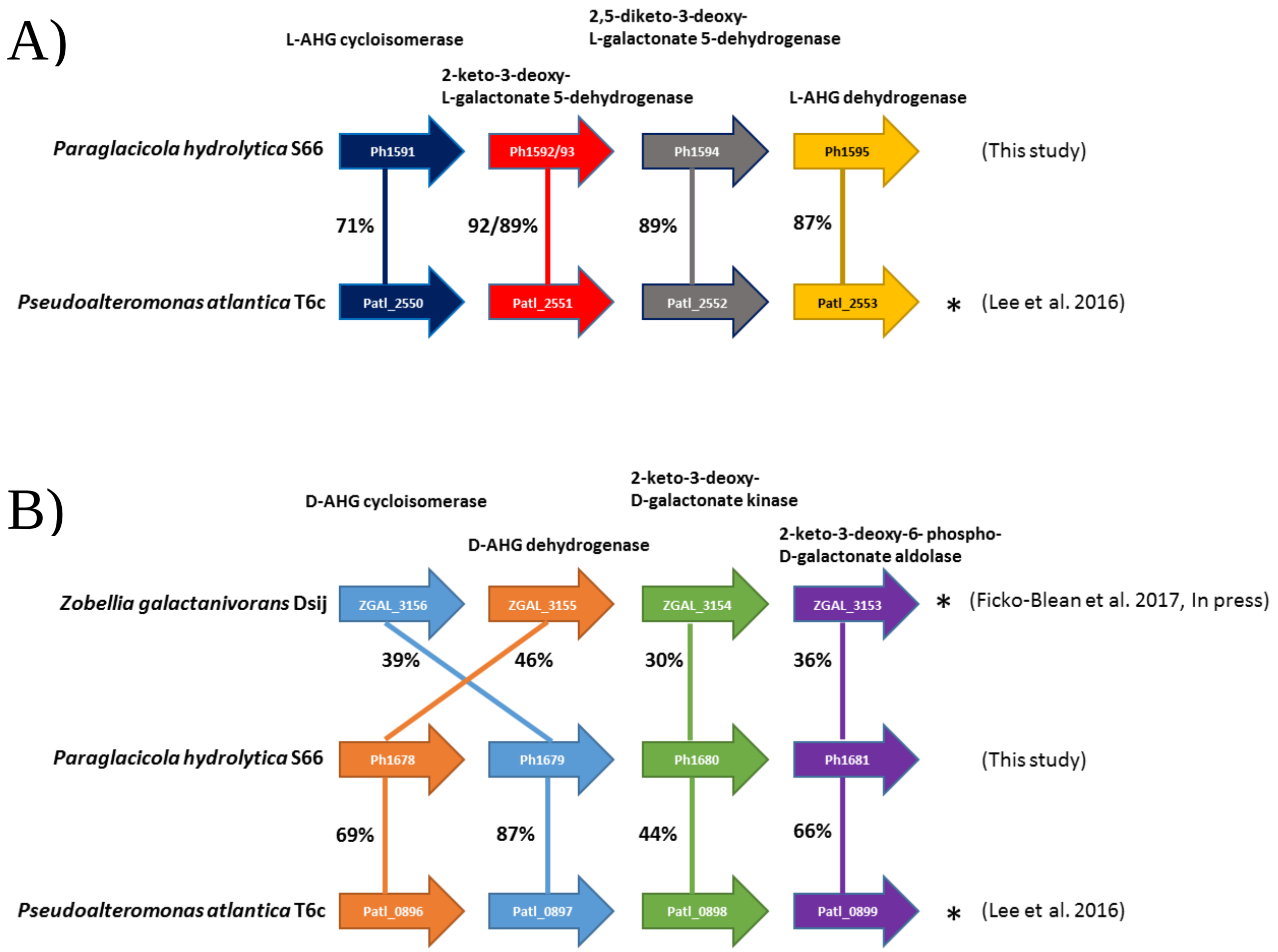

References

Ficko-Blean, E., Préchoux, A., Thomas, F., Rochat, T., Larocque, R., Zhu, Y., et al. (In Press). Carrageenan catabolism is encoded by a complex regulon in marine heterotrophic bacteria. *Nature Communications* doi: 10.1038/s41467-017-01832-6.

Lee, C.H., Kim, H.T., Yun, E.J., Lee, A.R., Kim, S.R., Kim, J.H., et al. (2014a). A novel agarolytic beta-galactosidase acts on agarooligosaccharides for complete hydrolysis of agarose into monomers. *Appl Environ Microbiol* 80(19)**,** 5965-5973. doi: 10.1128/AEM.01577-14.

Lee, S.B., Cho, S.J., Kim, J.A., Lee, S.Y., Kim, S.M., and Lim, H.S. (2014b). Metabolic pathway of 3,6-anhydro-L-galactose in agar-degrading microorganisms. *Biotechnology and Bioprocess Engineering* 19(5)**,** 866-878. doi: 10.1007/s12257-014-0622-3.

Lee, S.B., Kim, J.A., and Lim, H.S. (2016). Metabolic pathway of 3,6-anhydro-D-galactose in carrageenan-degrading microorganisms. *Applied Microbiology and Biotechnology* 100(9)**,** 4109-4121. doi: 10.1007/s00253-016-7346-6.

Prechoux, A., Genicot, S., Rogniaux, H., and Helbert, W. (2013). Controlling carrageenan structure using a novel formylglycine-dependent sulfatase, an endo-4S-iota-carrageenan sulfatase. *Mar Biotechnol (NY)* 15(3)**,** 265-274. doi: 10.1007/s10126-012-9483-y.

Prechoux, A., Genicot, S., Rogniaux, H., and Helbert, W. (2016). Enzyme-Assisted Preparation of Furcellaran-Like kappa-/beta-Carrageenan. *Mar Biotechnol (NY)* 18(1)**,** 133-143. doi: 10.1007/s10126-015-9675-3.
